# Self-Association and Microhydration of Phenol: Identification of Large-Amplitude Hydrogen Bond Librational Modes

**DOI:** 10.3390/molecules29133012

**Published:** 2024-06-25

**Authors:** Dmytro Mihrin, Karen Louise Feilberg, René Wugt Larsen

**Affiliations:** 1 Department of Chemistry, Technical University of Denmark, Kemitorvet 206, 2800 Kongens Lyngby, Denmark; 2 DTU Offshore, Technical University of Denmark, Elektrovej 375, 2800 Kongens Lyngby, Denmark; klfe@dtu.dk

**Keywords:** phenol cluster molecules, vibrational spectroscopy, neon matrices, large-amplitude librational motion, hydrogen bonding, London dispersion forces, local energy decomposition

## Abstract

The self-association mechanisms of phenol have represented long-standing challenges to quantum chemical methodologies owing to the competition between strongly directional intermolecular hydrogen bonding, weaker non-directional London dispersion forces and C–H⋯π interactions between the aromatic rings. The present work explores these subtle self-association mechanisms of relevance for biological molecular recognition processes via spectroscopic observations of large-amplitude hydrogen bond librational modes of phenol cluster molecules embedded in inert neon “quantum” matrices complemented by domain-based local pair natural orbital-coupled cluster DLPNO-CCSD(T) theory. The spectral signatures confirm a primarily intermolecular O-H⋯H hydrogen-bonded structure of the phenol dimer strengthened further by cooperative contributions from inter-ring London dispersion forces as supported by DLPNO-based local energy decomposition (LED) predictions. In the same way, the hydrogen bond librational bands observed for the trimeric cluster molecule confirm a pseudo-*C*_3_ symmetric cyclic cooperative hydrogen-bonded barrel-like potential energy minimum structure. This structure is vastly different from the sterically favored “chair” conformations observed for aliphatic alcohol cluster molecules of the same size owing to the additional stabilizing London dispersion forces and C–H⋯π interactions between the aromatic rings. The hydrogen bond librational transition observed for the phenol monohydrate finally confirms that phenol acts as a hydrogen bond donor to water in contrast to the hydrogen bond acceptor role observed for aliphatic alcohols.

## 1. Introduction

Hydrogen bonding stands as a cornerstone in both the structure and function of supramolecular chemical interactions, governing the majority of biological recognition mechanisms and the properties of solvents, and guiding chemical reactivity. In intricate environments, highly directional hydrogen bonds represent just one among several other classes of non-covalent intermolecular interactions contributing to molecular association mechanisms. Non-directional attractive London dispersion forces constitute an integral component of every non-covalent interaction, contributing a substantial portion of the total association energy within any given molecular aggregate growing with the size of the molecular system. London dispersion forces become particularly important when the primary bond is weaker than typical conventional hydrogen bonds, such as those between O-H⋯N and O-H⋯O motifs [1]. In addition, secondary interactions such as non-conventional O-H⋯π hydrogen bonds and even weaker C-H⋯π contacts often compete effectively with conventional hydrogen bonds in aromatic molecular systems [2,3]. The complex interplay between London dispersion, electrostatic forces, and weak donor-acceptor interactions represents a significant theoretical challenge for predictive ab initio methods targeting realistic supramolecular assemblies. Currently, only high-level post-Hartree-Fock ab initio quantum chemistry approaches such as coupled cluster theory CCSD(T) [4,5,6] consistently provide accurate predictions of non-covalent interactions, particularly in the presence of extensive van der Waals forces. However, the computational cost of these advanced ab initio methods scales rapidly, rendering them impractical for larger molecular systems, whereas lower-tier methods such as density functional theory (DFT), despite reliance on empirical corrections, often fail to achieve the necessary chemical accuracy [7]. The prototypical homocluster molecules of phenol (PhOH) have been investigated extensively and have earned a place in benchmark databases for biomolecular systems [8], although the computational costs for theoretical CCSD(T) predictions become prohibitive already at the (PhOH)_2_ level due to the size of the aromatic fragments [9,10]. The introduction of these aromatic structures facilitates the formation of intermolecular contacts involving the π-electron clouds. The self-association mechanisms of phenol thus involve an interplay of both conventional primary O-H⋯O hydrogen bonds, substantial dispersive contributions and secondary C-H⋯π contacts constituting a perfect playground for the validation of lower-tier theoretical methodologies with accurate spectroscopic findings.

The currently available experimental spectroscopic data primarily consist of structural and vibrational observations for phenol dimer (PhOH)_2_ and phenol trimer (PhOH)_3_ as well as various phenol microhydrate cluster molecules PhOH·(H_2_O)_1-3_ [11,12,13,14,15,16,17,18,19,20]. While both microwave spectroscopy guided substitution structures and mid-IR transitions associated with strongly active intramolecular vibrational OH-stretching modes have been reported in the literature for these homo- and microhydrate cluster molecules, there are currently no reported observations of the low-energy transitions associated with the large-amplitude and highly anharmonic OH librational (hindered torsional) modes linked directly to these (cooperative) hydrogen bond networks. This class of large-amplitude hydrogen bond librational motion has been shown to be an accurate probe of the strength and directionality of the intermolecular hydrogen bonds [21,22,23]. For aliphatic alcohols, the alkyl groups in cyclic cluster molecules have previously been shown experimentally to avoid each other due to steric hindrance resulting in chair-like potential energy minima for alcohol trimers and “up-down-up-down” (relative to the hydrogen bond plane) minima for alcohol tetramers [24]. Furthermore, the spectroscopic detection of these large-amplitude hydrogen bond librational modes reveals the most dominating contributions to the change in vibrational zero-point energy upon complexation (ΔZPE) [25]. These observations help to translate high-level ab initio quantum chemical predictions of electronic equilibrium dissociation energies (*D*_e_) into accurate semi-empirical ground-state dissociation energies (*D*_0_), which is notoriously challenging from first principles quantum chemistry alone due to the highly anharmonic nature of this class of large-amplitude vibrational motion [26,27,28,29].

In the present work, we provide for the first time spectroscopic assignments for the experimentally less-accessible vibrational transitions associated with large-amplitude hydrogen bond librational motions for phenol dimer, phenol trimer and isotopic variants of the phenol monohydrate (PhOH·H_2_O/PhOH·D_2_O) embedded in inert cryogenic “quantum” neon matrices at 4 K. These experimental findings are complemented by systematic theoretical conformation searches and harmonic force field predictions employing both dispersion-corrected DFT and ab initio methodologies of the most stable potential energy minima. In addition, detailed analyses of the total interaction energies in these phenol cluster molecules are carried out by means of a local energy decomposition (LED) scheme to account for the competition between the different classes of intermolecular non-covalent forces.

## 2. Results

### 2.1. Phenol Cluster Molecules

**Figure 1:**
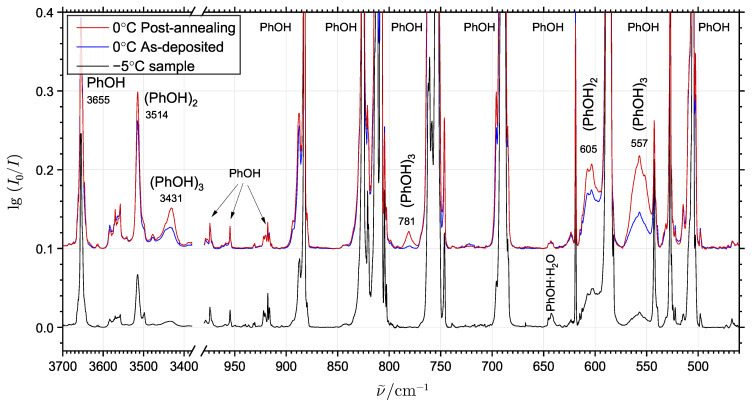
The infrared absorption spectra of phenol embedded in solid neon at 4 K. In the two different experiments shown, the phenol source sublimation vessel was thermostated at 0 °C (**bottom**) and at −5 °C (**top** and **bottom**, respectively) during deposition to achieve different mixing ratios with neon. The spectra of the 0 °C experiment were recorded before (blue trace) and after annealing of the matrix at 9.5 K (red trace). The spectra have been normalized to the phenol monomer transitions in the 650–575 cm^−1^ range. The experimentally observed transitions associated with the intramolecular OH-stretching and the large-amplitude hydrogen bond librational modes of (PhOH)_2_ and (PhOH)_3_ are indicated with the respective band origins.

Figure 1 shows the mid-infrared absorption spectra of doped neon matrices embedded with phenol at two different mixing ratios (by thermostating the phenol sublimation vessel at 0 °C and at −5 °C, respectively). In the former, most concentrated neon matrix experiment, a so-called pre-annealing spectrum (blue trace) was recorded immediately after the deposition of the sample and a post-annealing spectrum (red trace) was collected after the annealing procedure.

The primary distinct bands observed in the spectra above 3400 cm^−1^ are associated with the strongly IR-active intramolecular OH stretching modes of phenol monomer and the intensity-enhanced donor OH-stretching modes of phenol cluster molecules abundant in sub-% concentrations relative to the monomer. The bands observed at 3655 cm^−1^, 3515 cm^−1^ and 3431 cm^−1^ belong to the free OH-stretching mode of phenol monomer [11,13], the hydrogen-bonded donor OH-stretching modes of (PhOH)_2_ [11,13] and (PhOH)_3_ [11], respectively, in agreement with the observed concentration dependency, the effects of annealing and reported band origins from previous neon [11] and argon matrix isolation [14] and jet spectroscopy investigations [15,16]. The band origins for phenol monomer and phenol dimer embedded in neon have recently been revised with the latest observations at 3655 and 3515 cm^−1^, respectively [13]. The donor OH-stretching mode of (PhOH)_2_ has previously been observed at 3530 cm^−1^ in a jet REMPI investigation [15,16], whereas the dangling OH-stretching mode of (PhOH)_2_ and the free OH-stretching mode of the monomer have previously been assigned at 3654 cm^−1^ and 3657 cm^−1^ in jet expansions, respectively. Three transitions associated with the OH-stretching modes of (PhOH)_3_ have previously been assigned upon jet-cooling at 3394, 3441 and 3449 cm^−1^ suggesting a cyclic structure of (PhOH)_3_ [15], which has subsequently been confirmed by jet microwave spectroscopy [17,18,19].

**Figure 2:**
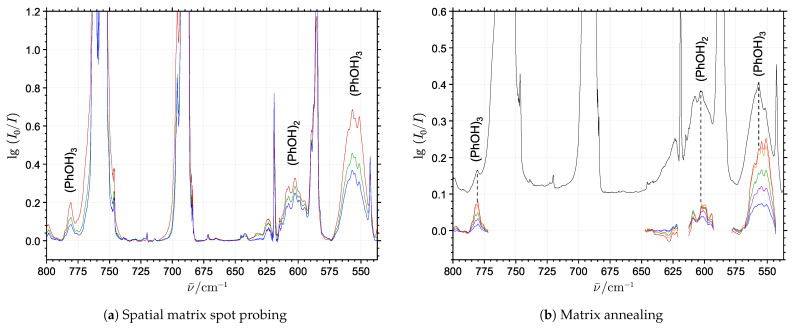
(**a**) The spectral dependence of the phenol/neon mixing ratio by spatial spot probing. The three shown spectra collected for spots with increasing phenol/ratio (the blue and red traces collected for the lowest and highest phenol/neon mixing ratios, respectively) have been normalized to the monomer transitions. The proposed dimer and trimer absorption features are differentiated based on their different growth rates relative to the monomer bands. (**b**) The evolution of the neon matrix spectra over time during annealing at 9.5 K. A series of difference spectra (annealing spectrum subtracted the pre-annealing spectrum) collected at 3 min intervals during the annealing procedure is shown. The colored difference spectra (the blue trace collected after 3 min and the red trace collected after 15 min, respectively) show excess absorption relative to the pre-annealing spectrum (black trace) demonstrating the progressive formation of phenol cluster molecules.

In the 400–800 cm^−1^ spectral range normally associated with the class of large-amplitude (hindered) torsional motions, several additional broad but still distinct bands, with intensities depending on both sample concentration and matrix annealing are observed. In order to distinguish the size of the cluster molecules, the concentration dependence and growth rate upon annealing of these bands were explored in additional experiments employing the phenol sublimation temperature in the 0–10 °C range during deposition. Figure 2a shows the spectral sampling of the doped neon matrix below 800 cm^−1^ at several different spatial locations limited to a diameter of approximately 3.5 mm of the infrared probe beam. This provides a variation of the local mixing ratio between neon and phenol molecules within the same matrix. The individual spectra have been normalized using the phenol monomer transition intensities in the 650–575 cm^−1^ range, which are not overlapped by the cluster molecule features. Figure 2b shows spectra below 800 cm^−1^ recorded before (black trace) and during annealing of the matrix at different steps (colored traces), with the latter calculated by subtracting the cold absorbance spectrum from the annealing spectra.

The experimental findings from the combined spatial spot probing and annealing of the doped neon matrices allow the identification of two different sets of bands belonging to phenol cluster molecules. The most distinct absorption feature below 800 cm^−1^, behaving as a cluster molecule band even for very low phenol/neon mixing ratios and always appearing in the spectra prior to annealing, is located at 605 cm^−1^. Its band intensity correlates well with the OH-stretching band at 3515 cm^−1^ and this transition is therefore assigned to the only large-amplitude hydrogen bond librational (strongly hindered OH torsional) fundamental expected for (PhOH)_2_. The two other distinct bands observed at 557 cm^−1^ and 781 cm^−1^ show higher stoichiometry compared to the assigned (PhOH)_2_ features. The most intense far-infrared band located at 557 cm^−1^ reveals the same concentration and annealing dependencies as the mid-infrared OH-stretching band at 3431 cm^−1^ assigned for (PhOH)_3_. The intensity of the band at 781 cm^−1^ similarly shows strong correlation with the 557 cm^−1^ and 3431 cm^−1^ bands with respect to the phenol mixing ratio and annealing procedures; however, in the high concentration experiments the spectral overlap with some broad satellite bands around 762 cm^−1^ impedes the exact correlations, tentatively assigned to CH out-of-plane bending motions of (PhOH)_2_/(PhOH)_3_. These exclusively experimental considerations suggest that the linked 557 cm^−1^ and 781 cm^−1^ bands should be assigned to transitions associated with large-amplitude hydrogen bond librational motion of the most stable conformation of (PhOH)_3_, which will be supported by complementary theoretical predictions in the following.

**Figure 3:**
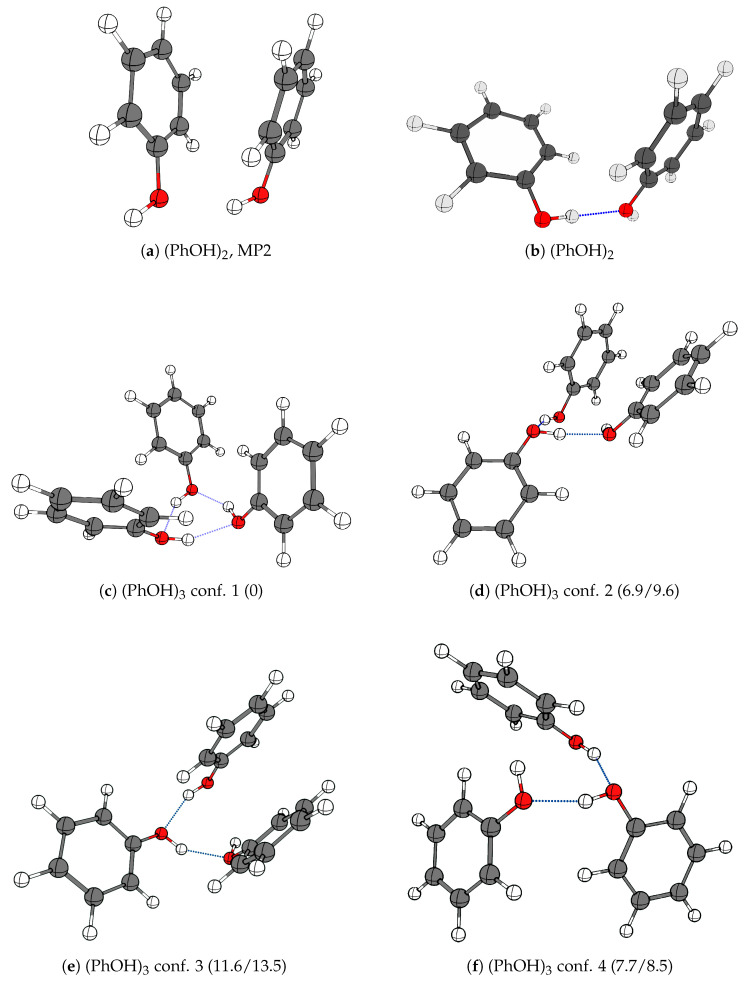
The optimized potential energy minima structures of the pure phenol cluster molecules (**a**) (PhOH)_2_ (MP2/AVQZ level), (**b**) (PhOH)_2_ (PW6B95-D4/ma-def2-QZVP level) and (**c**–**f**) the four different conformations of (PhOH)_3_. The relative zero-point energy corrected dissociation energies *D*_0_ (the change of zero-point energy (ΔZPE) calculated at PW6B95-D4/ma-def2-QZVP//SCS-MP2/AVQZ levels with electronic energies obtained at the DLPNO-CCSD(T)/AVQZ level, both values in kJ·mol^−1^) are given in brackets for each conformation.

Figure 3 shows the optimized molecular geometries of phenol and its hydrogen-bonded cluster molecules (PhOH)_2_ and (PhOH)_3_ using various theoretical methodologies, and provides the relative zero-point energy corrected ground-state dissociation energies for the four most stable conformations predicted for the (PhOH)_3_ system. The computational predictions of the molecular geometry for the (PhOH)_2_ system remain challenging even for modern computer resources. The MP2 quantum chemical method, which in general tends to provide consistent ab initio predictions for conventional hydrogen-bonded cluster molecules, runs into an issue in the treatment of the intermolecular interactions between the aromatic rings of the phenol fragments. Previously, a qualitatively correct structure has only been obtained with this methodology in conjunction with counterpoise corrections to account for the small basis set [10]. Considering the large basis set required to achieve accurate vibrational frequency predictions, this theoretical approach becomes prohibitively expensive. The optimized geometry of (PhOH)_2_ has been calculated using the MP2/AVQZ methodology (Figure 3a), which has shown significant underestimation of the hydrogen bonding in the complex, compared to what is observed in the experiment. Even with this relatively large basis set, the conventional MP2 method suffers from a strong over-binding effect between the aromatic rings, which in this case directly competes with the hydrogen bond. In order to achieve the desired accuracy for all cluster molecules investigated, the empirically scaled SCS-MP2 method and the dispersion-corrected hybrid PW6B95-D4 DFT functional have been selected, both known to perform comparatively well in the description of intermolecular non-covalent interactions [7]. These methods have reproduced experimental data with reasonable consistency; however, there is variance in the predicted dissociation energies and harmonic vibrational frequencies between the two different approaches.

The predicted harmonic frequencies of vibrational modes for the conformations of (PhOH)_2_, (PhOH)_3_ and PhOH·H_2_O are given in Table S1 in the Electronic Supplementary Information (ESI). For the (PhOH)_2_ system, for which only one conformation has been optimized (Figure 3b), both methods predict one highly IR-active transition where the band associated with the hydrogen bond librational mode is experimentally observed in the present work. For the even more challenging (PhOH)_3_ system, four different conformations have been optimized. Both methodologies show a large energy gap between the most stable cyclic pseudo-C_3_ symmetric barrel-like potential energy minimum structure involving three cooperative OH⋯O hydrogen bonds and the other three predicted energy minima (Figure 3c). The less stable conformations 2–4 differ in the arrangements of the aromatic rings, or substitute the conventional intermolecular OH⋯O hydrogen bonds for the non-conventional OH⋯π bond or the stacking of aromatic rings. The differences in the optimized geometries result in different rankings of the dissociation energy calculated for the less stable conformations (Figure 3d,f).

**Figure 4:**
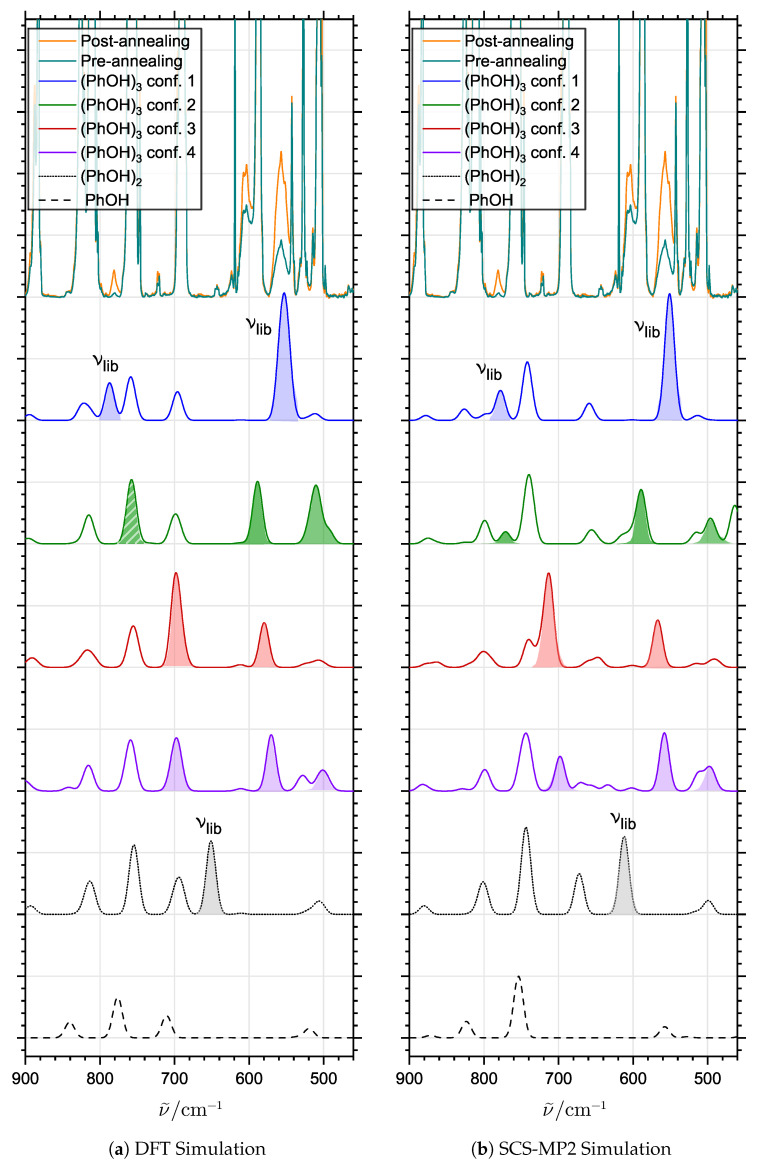
The simulated vibrational spectra of PhOH, (PhOH)_2_ and the predicted conformations of (PhOH)_3_ using the DFT (PW6B95-D4) and SCS-MP2 methodologies. Harmonic vibrational mode frequencies have been scaled separately using a scaling factor of 0.95 for the large-amplitude hydrogen bond librational modes and 0.97 for less perturbed intramolecular transitions. The predicted large-amplitude hydrogen bond librational transitions are marked with filled areas. The striped area indicates spectral overlap between a librational transition and a less perturbed intramolecular transition. The band positions for the experimentally assigned librational transitions of (PhOH)_2_ and (PhOH)_3_ are indicated on the trace for the identified conformation. The experimental pre- and post-annealing spectra are provided above the simulations.

**Figure 5:**
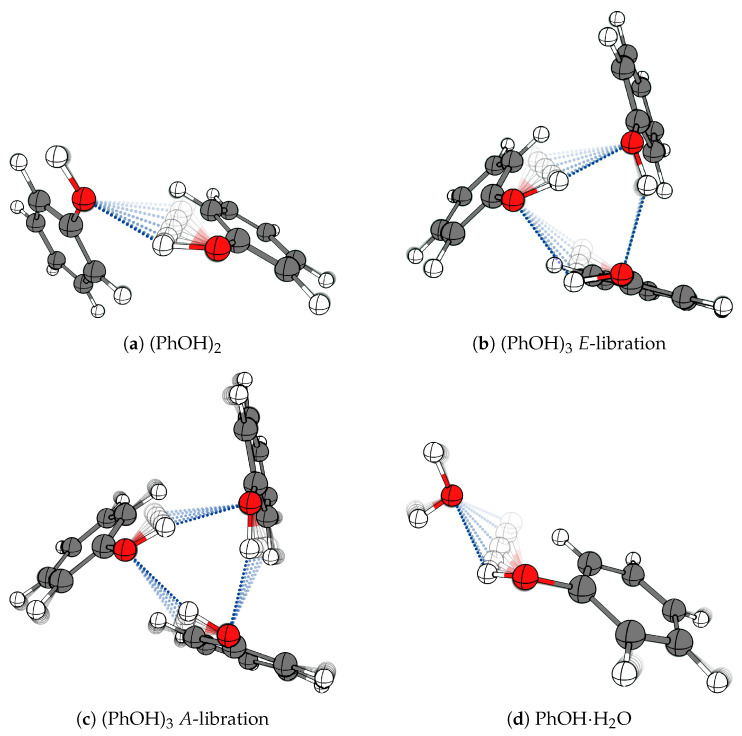
Animations of the large-amplitude hydrogen bond librational modes of (**a**) (PhOH)_2_, (**b**,**c**) (PhOH)_3_ and (**d**) PhOH·H_2_O associated with the experimentally assigned absorption bands.

The simulated vibrational spectra for the four different conformations of (PhOH)_3_ are shown in Figure 4. In this simulation, the calculated harmonic frequencies of the normal modes have been scaled using a separate factor for the modes involving large-amplitude hindered torsional motion of the OH-groups, which are estimated from the experimental spectra. The predicted pseudo-C_3_-symmetric global potential energy minimum (conf. 1) has two near-degenerate highly IR-active librational modes (Figure 5b) estimated around 550 cm^−1^. Owing to the pseudo-C_3_ symmetry, there are no other predicted intense transitions associated with hydrogen bond librational modes in the 500–700 cm^−1^ range, unlike the asymmetric conformations 2–4, which all have at least two distinct IR-active hydrogen bond librational transitions in this region (indicated by filled colored band areas). In the case of conformation 2, one of the predicted hydrogen bond librational transitions has severe spectral overlaps with other less perturbed intramolecular transitions (DFT simulation); however, the two remaining highly IR-active hydrogen bond librational transitions should still be observed clearly between 500 and 600 cm^−1^ for this conformation, which is not supported in the present experiments. The second distinct transition assigned experimentally for (PhOH)_3_ in the present experimental work at 781 cm^−1^ is also supported by the DFT and SCS-MP2 quantum chemical predictions. While the positions of the intramolecular transitions seem better predicted by the DFT approach, both methods agree on the position of the third IR-active transition associated with the concerted hydrogen bond librational motion of the most stable C_3_ conformation of (PhOH)_3_ (Figure 5c), involving all three O-H⋯O motifs, which is close to the experimental value. The relative transition intensity ratio between the two hydrogen bond librational transitions at 557 and 781 cm^−1^ of (PhOH)_3_ is also qualitatively reproduced, although the overlap with the transitions attributed with perturbed intramolecular out-of-plane CH bending modes of (PhOH)_2_ and (PhOH)_3_ at higher concentrations makes more accurate analyses impossible. Together with the near-degenerate donor OH-stretching transitions observed at 3431 cm^−1^, the two transitions associated with hydrogen bond librational motion allow us to unambiguously conclude that only the most stable pseudo-C_3_ barrel-like conformation is observed in the present neon matrix experiments, both in the lower concentration regime and after being formed via annealing under cryogenic conditions. This is in strong contrast to previous findings for cyclic trimers of aliphatic alcohols, which have been shown to form “chair” structures due to steric hindrance [24,30].

### 2.2. The Phenol Monohydrate

**Figure 6:**
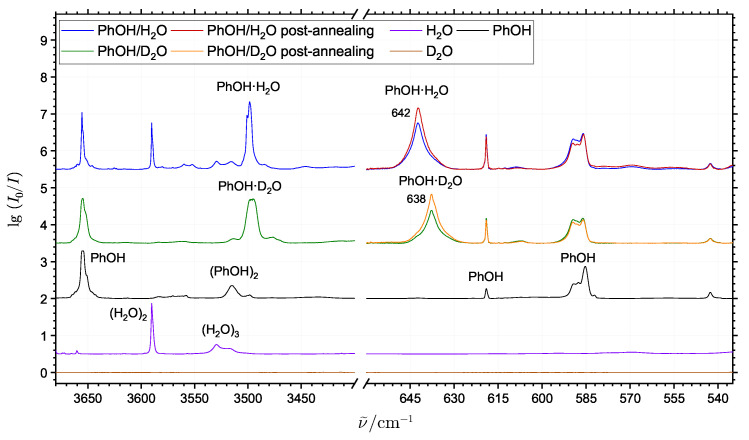
The infrared absorption spectra of phenol (black trace, small traces of H_2_O), H_2_O (purple trace) and D_2_O (brown trace) together with spectra of phenol/H_2_O (blue trace) and phenol/D_2_O (green trace) mixtures. For the two mixtures the spectra after annealing of the matrix to 9.5 K (red and orange traces, respectively) are shown below 650 cm^−1^. The new assigned transitions associated with the large-amplitude hydrogen bond librational modes of the PhOH·H_2_O and PhOH·D_2_O monohydrates are indicated in the spectra with their respective band positions.

**Figure 7:**
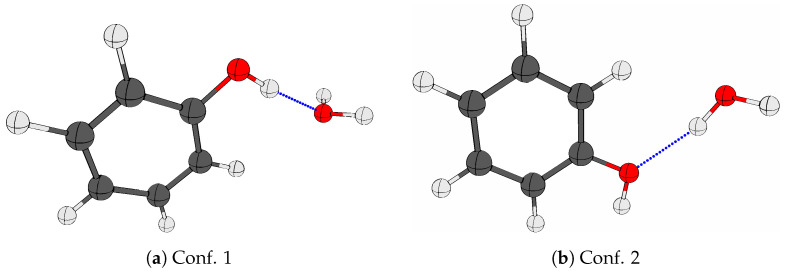
The optimized global (**a**) and local (**b**) potential energy minima structures of the phenol monohydrate (PhOH·H_2_O) employing the SCS-MP2/AVQZ level of theory.

Figure 6 shows the spectra collected for neon matrices doped with pure phenol, pure H_2_O and pure D_2_O together with spectra of phenol/H_2_O and phenol/D_2_O mixtures in the OH-stretching range (3400–3700 cm^−1^) and the relevant range for the transitions associated with large-amplitude hydrogen bond librational motion (500–670 cm^−1^). In between the hydrogen-bonded OH-stretching transitions for (PhOH)_2_, (H_2_O)_2_ and (H_2_O)_3_, it is evident that the simultaneous deposition of phenol and H_2_O reveal a new strongly IR-active distinct band at 3499 cm^−1^, which has recently been assigned to the phenol monohydrate PhOH·H_2_O [13]. The corresponding neon matrix isolation experiments for phenol/D_2_O show that the assigned signal is indeed due to most stable conformation of this mixed cluster molecule, as the band shifts only slightly (and broadens somewhat due to spectral overlap with traces of the phenol-HDO complex), whereas the less stable conformation with water as hydrogen bond donor to phenol would absorb in the entirely different OD stretching region. This is supported by the quantum chemical results showing that the predicted zero-point energy corrected dissociation energy *D*_0_ of the global potential energy minimum (Figure 7a) is 9 kJ·mol^−1^ higher than predicted for the local minimum (Figure 7b). The donor OH-stretching transitions of the phenol hydrates PhOH·(H_2_O)_1-3_ have previously been observed in jet expansions at 3524 cm^−1^, 3388 cm^−1^ and 3345 cm^−1^ [20], respectively, and the OH-stretching transition of the phenol monohydrate embedded in argon matrices has been assigned at 3461 cm^−1^ [14].

In the spectral region below 700 cm^−1^, a highly IR-active distinct band is observed at 642 cm^−1^ in the neon matrices doped simultaneously with phenol and H_2_O. Furthermore, the band intensity responds significantly to annealing, indicating complex formation events in the neon matrix. The band shifts slightly down to 638 cm^−1^ in the experiment where phenol is deposited together with D_2_O. The small isotopic shift of this transition confirms the proposed structure of the phenol monohydrate PhOH·H_2_O where the phenol molecule acts as the hydrogen bond donor and the observed transition is straightforwardly assigned to the large-amplitude donor librational motion of PhOH·H_2_O as visualized in Figure 5d. We do not observe any features in the low-energy part of the spectrum which could be clearly attributed to the less stable conformation.

### 2.3. Supporting Theoretical Analysis

The results of the LED analyses for the phenol cluster molecules PhOH·H_2_O, (PhOH)_2_ and (PhOH)_3_ are summarized in Table 1. The optimized potential energy minima structures obtained from the PW6B95-D4/ma-def2-QZVP and SCS-MP2/AVTZ methodologies were used for the subsequent LED analyses at the DLPNO-CCSD(T)/AVQZ level of theory. The electronic equilibrium dissociation energies *D*_e_ and the zero-point corrected dissociation energies *D*_0_ obtained from the higher DLPNO-CCSD(T) level on the optimized geometries are denoted *D*_e_(CC) and *D*_0_(CC), respectively. In the case of the smaller (PhOH)_2_ and PhOH·H_2_O systems, geometry optimizations were also performed using the highest feasible SCS-MP2/AVQZ level to access the potential influence of the basis set size. The basis set does not have a significant influence on the dissociation energies for (PhOH)_2_ as seen from the minor 0.3 kJ·mol^−1^ (0.1 %) increase in *D*_e_(CC), when calculated on the optimized geometry employing the larger AVQZ basis set. However, the molecular properties obtained directly from the method, specifically the value of change in zero-point energy upon complexation ΔZPE, is much more dependent on the basis set. In that respect, the cheaper DFT approach performs better for (PhOH)_2_, likely due to lower basis set requirements. In general, when approaching the basis set limit, the SCS-MP2 method tends to underestimate non-covalent interactions, as can be seen from the results for the PhOH·H_2_O system. Still, the SCS-MP2 wave function approach provides more consistent results when dealing with the larger (PhOH)_3_ conformations, when evaluated against the high-level DLPNO-CCSD(T) energies based on the same respective optimized molecular geometries probably due the larger contributions from London dispersion forces.

The interfragment dispersion energies, obtained from the LED analysis, highlight the primary differences between the intermolecular forces dominating in the PhOH·H_2_O and the (PhOH)_2_ systems. Both for the DFT and the SCS-MP2 approach, the resulting electronic equilibrium and zero-point energy corrected dissociation energies are almost identical for the global potential energy minima conformations. However, whereas the interfragment dispersion energy of (PhOH)_2_ constitutes as much as 72% of the *D*_e_-value, this contribution constitutes only 29% for the less electron-rich PhOH·H_2_O system.

While the increase in the basis set size in the calculated SCS-MP2 model of (PhOH)_2_ leads to only a minor change in the cumulative value of *D*_e_, it causes over 2 kJ·mol^−1^ difference in the dispersion energy, suggesting a noticeable change in the predicted balance of intermolecular forces and the strong competition with the hydrogen bond. When comparing the predictions for the (PhOH)_2_ to (PhOH)_3_ systems with the AVTZ basis, the interfragment dispersion energy of the (PhOH)_3_ system is lower by 1.3 kJ·mol^−1^, and the proportion of *E*_disp_ in the total dissociation energy is also lowered to 67%. The distance between the aromatic rings increases, e.g., the calculated interatomic distance between C-atoms in the *para*-positions increases from 6 to 6.25 Å. Despite the cooperative effect, the hydrogen bond length increases from 1.93 to 1.98 Å, while at the same time due to mutual rotation of the rings, the hydrogen in the *meta*-position becomes oriented towards the π-cloud of the adjacent fragment. The same can be observed for the DFT geometries. The DFT structures overall show significantly lower weighting of the dispersive interactions for the (PhOH)_2_ and (PhOH)_3_ structures.

The effect of hydrogen bond cooperativity is common for cyclic cluster molecules and can be probed experimentally by the relative average hydrogen bond librational energy compared to the single hydrogen bond librational transition energy of the respective dimer [24,30,33]. In the case of (PhOH)_3_, we observe experimental evidence of cooperativity in the larger average observed hydrogen bond librational energy of 631 cm^−1^ (assuming the assigned 557 cm^−1^ transition is degenerate) relative to the observed librational band at 605 cm^−1^ for (PhOH)_2_. It is worth noting that only the high-level wavefunction-based methods properly capture this effect. The DFT approach predicts an almost exactly three-times larger dissociation energy for the (PhOH)_3_ system with three cooperative intermolecular hydrogen bonds relative to the dissociation energy of (PhOH)_2_. The high-level DLPNO-CCSD(T) predictions of the DFT optimized molecular structures capture the experimental findings that *D*_0_((PhOH)_3_) > 3 × *D*_0_((PhOH)_2_) and so does the SCS-MP2 approach even before the DLPNO-CCSD(T) refinement.

While we cannot quantitatively isolate the CH⋯π interactions using the LED approach, we can roughly estimate the relative roles of this contact between the phenol cluster molecules via selected structural parameters. In the (PhOH)_2_ system, the O-H⋯O angle is 127°, and the planes of the aromatic rings are oriented near perpendicular around the hydrogen bond axis. The bond angle in the (PhOH)_3_ system is 90°. The structure of (PhOH)_4_ was calculated using the DFT approach to extend this series (see ESI), and the bond angle is 103° for this system. The CH⋯π interaction is estimated as a function of the distance from the hydrogen in the *ortho*-position to the center of the adjacent phenol fragment. This distance is 3.75 Å, 3.30 Å and 3.37 Å for (PhOH)_2-4_, respectively. The relatively unobstructed rotation of the rings in the dimer results in optimal orientation that does not benefit a strong CH⋯π contact. In the (PhOH)_3_ and (PhOH)_4_ systems, the cooperative hydrogen bond networks constrict the mutual orientation of the rings, and therefore the hydrogen bond angles become smaller, but this orientation favors the CH⋯π interaction. The CH⋯π bond distance is shorter in (PhOH)_3_, but then increases again when transitioning to (PhOH)_4_. This appears to be due to strengthening of the cooperative hydrogen bond network resulting in an opening of the phenyl ring “barrel”-like structure.

## 3. Materials and Methods

### 3.1. Experimental Details

Phenol powder (Sigma-Aldrich, Søborg, Denmark, ≥99.5%) was purified by several cycles of vacuum sublimation over pre-baked molecular sieves (4Å) to eliminate residual air and moisture. Milli-Q grade regular water and isotopically enriched D_2_O (Sigma-Aldrich, Søborg, Denmark, 99% D-atoms) samples were degassed under vacuum.

The experimental setup consists of a Bruker Vertex 80V Fourier-transform spectrometer used with a DE-204 4 K (Advanced Research Systems, Inc., Macungie, PA, USA) closed-cycle cryocooler. A transmission sample holder with a wedged diamond cold window (Diamond Materials, GmbH, Freiburg im Breisgau, Germany) which can be cooled to 4.5–5.0 K is mounted onto the cold plate of the cooler. The cryocooler is surrounded by a rotatable vacuum shroud equipped with two wedged diamond windows and a gate valve allowing the insertion of the inlet system into the vacuum space of the cryostat. The wedged diamond windows minimize spectral interference fringes from internal reflections in the windows. The optical setup of the spectrometer consisted of a globar radiation source, a germanium-coated KBr beam splitter together with a broad-band HgCdTe detector for the complete infrared spectral range (4000–450 cm^−1^). A spectral resolution of 0.6 cm^−1^ was used throughout the measurements.

The inert gas matrix is obtained by simultaneous deposition of neon gas (99.999%, Air Liquide Danmark A/S, Taastrup, Denmark) with vapors of the samples through separate inlet tubes, which are brought to within 5 mm of the cold sample window using a motorized stage. The neon is supplied from an MKS G-series mass flow controller, passing a 1 m-long LN_2_-cooled coiled trap for impurities, at a flow rate of 6–8 sccm depending on the desired mixing ratio. Water vapor was supplied from a variable-pressure (30–80 Pa) supply volume through a small-flow Swagelok metering valve, and phenol vapor was deposited directly from the sublimation vessel thermostated in the temperature range −5–10 ± 0.2 °C throughout the deposition process.

Following acquisition of the initial (“pre-annealing”) matrix spectra, the samples were annealed by raising the temperature to 9 K for 60 min using a LakeShore controller operating a resistive heater and a Si-diode temperature sensor attached to the cold head. Subsequently, the heater was deactivated, allowing the matrix to cool again to 4–5 K before recording the “post-annealing” spectra. This annealing procedure softens the neon matrix and allows the diffusion of phenol molecules within the matrix and triggers the self-association of phenol into hydrogen-bonded cluster molecules.

The deposition of the samples via separate inlet tubes creates intentional spatial inhomogeneity in the phenol/water/neon mixing ratio, which is exploited to enable spatial “spot-to-spot” matrix probing and differentiate between cluster sizes in the same experiment. The effective diameter of the probed region of the matrix is comparable to the employed 3.5 mm aperture size. The background spectra were all collected of the evacuated cryostat at room temperature.

### 3.2. Computational Details

The molecular geometries of the phenol cluster molecules were predicted using the CREST (2.12, Bonn, Germany) [34] conformational search utility employing the GFN2-xTB methodology [35]. The 20 most stable structures suggested by the utility for each cluster molecule were then further optimized using several quantum chemical methods in ORCA (5.0.4, Max-Planck-Institut für Kohlenforschung, Germany) [36]: the MP2 [37] and SCS-MP2 [38] approaches with aug-cc-pV*n*Z [39] (*n* = T, Q) basis sets (denoted AVTZ, AVQZ), and the hybrid dispersion-corrected PW6B95-D4 [40,41,42] functional with the ma-def2-QZVP basis set [43,44] (later denoted as the DFT method). The optimized geometries of the most stable structures were then used for the respective harmonic vibrational frequencies and the dissociation energy calculations.

The electronic equilibrium dissociation energies (*D*_e_) of the cluster molecules were obtained by performing domain-based local pair natural orbital coupled cluster (DLPNO-CCSD(T)) [45,46] single-point calculations on the respective DFT and SCS-MP2 geometries. The cut-offs for the DLPNO methodology were set by the TightPNO keyword [47]. The zero-point corrected ground-state dissociation energies (*D*_0_) of the cluster molecules were calculated as the sum of the DLPNO-CCSD(T) electronic dissociation energies (*D*_e_) and the ΔZPE values from the respective base calculations.

The intermolecular London dispersion energies of the cluster molecules were extracted using the DLPNO-based local energy decomposition (LED) scheme [31]. The procedure is described in the literature [31,32].

The resolution of the identity approximation was used in all of the calculations with the appropriate fitting basis sets [48,49,50,51]. The numerical “chain-of-spheres” approximation (COSX [52,53]) was used with the MP2 level calculations. The calculations were all carried out using the DTU Computing Center (DCC) cluster [54].

## 4. Conclusions

The self-association and microhydration molecular recognition mechanisms of phenol have been explored experimentally by means of the less accessible vibrational transitions associated with large-amplitude hydrogen bond librational motion. The present work provides for the first time unambiguous spectroscopic assignments for the large-amplitude and highly anharmonic hydrogen bond librational modes of the phenol dimer (605 cm^−1^), the phenol trimer (557 and 781 cm^−1^) and two isotopic variants of the phenol monohydrate (642 cm^−1^ for PhOH·H_2_O and 638 for PhOH·D_2_O) embedded in inert cryogenic “quantum” neon matrices at 4 K. While accurate microwave spectroscopic information concerned with the molecular structures of these systems has been reported in the literature previously, these new experimental findings provide important constraints for future high-level quantum chemical models of the corresponding intermolecular potential energy surfaces.

The experimental findings have been complemented by extensive conformation search sampling and further computational optimizations employing both dispersion-corrected DFT and wavefunction ab initio methodologies of the most stable potential energy minima. In addition, the local energy decompositions of the total interaction energies for these phenol cluster molecules have been extracted. The local energy decompositions reveal an interplay of several binding mechanisms and active sites in the phenol cluster molecules resulting in a complex internal competition of non-covalent forces. The pure cluster molecules of phenol were found to exhibit OH⋯O cooperative hydrogen bonds, CH⋯π contacts and strong competing London dispersion forces between the aromatic rings. The larger phenol cluster molecules were calculated to have larger relative non-dispersive energy components compared to phenol dimer.

The accurate determination of the molecular geometries and intermolecular potential energy surfaces of these complexes is still challenging for the currently feasible levels of theory, which for systems of this size are limited to *meta*-GGA DFT or MP2 approaches with incomplete basis sets. The inconsistency between some of the energy parameters predicted by the methodologies tested here highlights the necessity of the inclusion of the *state-of-the-art* electronic structure methods in order to obtain reliable ab initio descriptions of these prototypical phenol cluster molecules. 

## Figures and Tables

**Table 1:** The electronic equilibrium dissociation energies (*D*_e_) for PhOH·H_2_O, (PhOH)_2_ and (PhOH)_3_ calculated both directly using the theoretical approaches PW6B95-D4/ma-def2-QZVP (denoted as DFT) and SCS-MP2 and the higher DLPNO-CCSD(T)/AVQZ level (*D*_e_ (CC)) on the respective optimized geometries, the change of zero-point energy (ΔZPE), the zero-point energy corrected dissociation energies (*D*_0_, *D*_0_(CC)), the interfragment dispersion energies (Δ*E*_disp, frag_) and the sums of the dispersive (∑*E*_disp_) and non-dispersive parts of *D*_e_ (∑*E*_N/D_, given as *D*_e_(CC)−∑*E*_disp_ [31,32]). The SCS-MP2 results for PhOH·H_2_O are obtained using the AVQZ basis set. All values are given in units of kJ·mol^−1^.

	DFT							SCS-MP2						
	PhOH·H_2_O		(PhOH)_2_	(PhOH)_3_				PhOH·H_2_O		(PhOH)_2_	(PhOH)_3_			
	Conf. 1	Conf. 2		Conf. 1	Conf. 2	Conf. 3	Conf. 4	Conf. 1	Conf. 2		Conf. 1	Conf. 2	Conf. 3	Conf. 4
*D*_e_	27.9	18.3	27.8	76.3	74.4	71.7	73.1	26.6	17.8	31.7	95.5	85.4	85.2	87.0
*D*_e_(CC)	28.9	19.8	30.0	89.2	80.8	78.0	81.6	28.8	19.7	30.1	90.1	81.7	78.5	82.4
											* 30.4			
*D*_0_	20.9	11.7	22.5	66.6	63.2	61.0	62.4	19.7	11.6	24.5	79.6	70.6	71.2	71.9
*D*_0_(CC)	21.8	13.2	24.8	79.5	69.6	67.3	70.9	22.0	13.6	23.0	74.3	66.9	64.5	67.3
ΔZPE	7.1	6.5	5.2	9.7	11.2	10.7	10.7	6.9	6.2	7.2	15.8	14.7	14.0	15.1
									* 4.2					
Δ *E*_disp, frag_	8.5	9.1	17.8	15.7	25.6	27.0	18.6	8.3	8.5	21.8	20.5	26.3	31.8	20.8
				15.9	12.6	17.5	14.3				20.1	12.9	18.5	17.9
				15.9	12.2	15.5	13.1				20.1	12.5	16.9	14.1
∑*E*_disp_	8.5	9.1	17.8	47.6	50.4	59.9	46.0	8.3	8.5	21.8	60.7	51.6	67.2	52.8
											* 19.7			
∑*E*_N/D_	20.4	10.7	12.3	41.7	30.5	18.1	35.7	20.6	11.3	8.3	29.4	30.0	11.3	29.6
											* 10.6			

* Optimized geometries and harmonic vibrational frequencies predicted at the SCS-MP2/aug-cc-pVQZ level.

## Data Availability

Data are contained within the article and Supplementary Materials.

## Supplementary Materials

The following supporting information can be downloaded at https://www.mdpi.com/article/10.3390/molecules29133012/s1. The material contains the optimized potential energy minima geometries of the (PhOH)_1-4_ and PhOH·H_2_O cluster molecules in XYZ format and the predicted harmonic vibrational mode frequencies for the respective structures.
