# Supplementary material for: Self-Association and Microhydration of Phenol: Identification of Large-Amplitude Hydrogen Bond Librational Modes
Source: Molecules. 2024 Jun 25;29(13):3012. doi: 10.3390/molecules29133012 (PMC11243154; doi:10.3390/molecules29133012)
Supplement: Supplementary file 1 [file molecules-29-03012-s001.zip › molecules-3065565-supplementary.pdf]

Self-Association Mechanisms of Phenol: Identification of Large-Amplitude Hydrogen Bond Librational Modes - Supplementary Materials

Dmytro Mihrin[1,2] , Karen Louise Feilberg[2] and René Wugt Larsen[1]

[1]Department of Chemistry, Technical University of Denmark, Kemitorvet 206, 2800 Kgs. Lyngby, Denmark;

[2]DTU Offshore, Technical University of Denmark, Elektrovej 375, 2800 Kgs. Lyngby, Denmark *Correspondence: rewl@kemi.dtu.dk

2024

Contents

List of Figures **3**

List of Tables **4**

1 Introduction **5**

1.1 Structures . . . 6

1.1.1 XYZ coordinates (PW6B95/ma-def2-QZVP, Å) . . . 6

1.1.2 XYZ coordinates (SCS-MP2, Å) . . . 8

1.2 Calculated vibrational frequencies . . . 12

List of Figures

S1 The calculated global minimum of the $(PhOH)_4$ complex using the PW6B95/ma-def2-QZVP level of theory. 6

List of Tables

S1 The selected calculated IR-active harmonic vibrational frequencies (ω, cm^{-1}), located in spectral ranges of interest, and their intensities (I, km·mol^{-1}) using the DFT (PW6B95-D4/ma-def2-QZVP) and the SCS-MP2/aug-cc-pVTZ levels of theory, and the observed band positions for the assigned modes (**exp**, cm^{-1}). Conformation indices are given in parentheses and correspond to the structures in Figure 3 of the main text. 12

S2 The calculated harmonic vibrational frequencies (ω, cm^{-1}) and intensities (I, km/mol) of phenol (PhOH) and phenol dimer $(PhOH)_2$ using the SCS-MP2 method on aug-cc-pVTZ (ATZ) and aug-cc-pVQZ (AQZ) basis sets. 12

S3 The calculated harmonic vibrational frequencies (ω, cm^{-1}) and intensities (I, km/mol) of the phenol trimer $(PhOH)_3$ clusters using the SCS-MP2 method on aug-cc-pVTZ (ATZ) basis set. 15

S4 The calculated harmonic vibrational frequencies (ω, cm^{-1}) and intensities (I, km/mol) of phenol (PhOH) and phenol dimer $(PhOH)_2$ using the PW6B95-D4 method on ma-def2-QZVP basis set. 18

S5 The calculated harmonic vibrational frequencies (ω, cm^{-1}) and intensities (I, km/mol) of the phenol trimer $(PhOH)_3$ clusters using the PW6B95-D4 method on ma-def2-QZVP basis set. 21

Chapter 1

Introduction

The following sections provide supplementary information on the results from quantum chemical calculations. Calculations were carried out in ORCA package using two computational methods: 1) PW6B95-D4/ma-Def2-QZVP and 2) SCS-MP2/aug-cc-VTZ/aug-cc-VQZ.

The supplement contains:

- Visualisation of the global minimum for the phenol tetramer system, obtained from the DFT calculation.
- XYZ cartesian coordinates of the optimised structures of phenol complexes investigated in the publication, employing both methods.
- Full tables of the harmonic vibrational frequencies predicted for the respective minima structures with intensities.

1.1 Structures

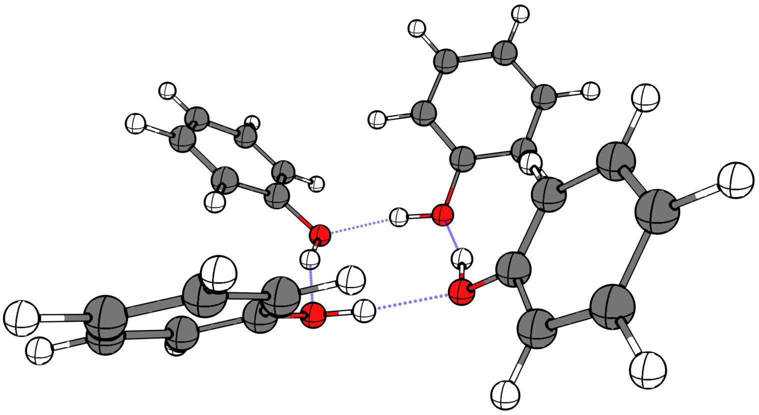

Figure S1: The calculated global minimum of the $(PhOH)_4$ complex using the PW6B95/ma-def2-QZVP level of theory.

1.1.1 XYZ coordinates (PW6B95/ma-def2-QZVP, Å)

PhOH.

C	1.206373000	0.248107000	0.000000000
C	1.207852000	-1.132185000	0.000000000
C	0.000903000	0.929818000	0.000000000
C	-1.193707000	0.229406000	0.000000000
C	-1.179182000	-1.153244000	0.000000000
C	0.019038000	-1.842057000	0.000000000
O	0.046772000	2.286668000	0.000000000
H	-0.842332000	2.637973000	0.000000000
H	2.124314000	0.810708000	0.000000000
H	2.148410000	-1.657823000	0.000000000
H	-2.130798000	0.765578000	0.000000000
H	0.027621000	-2.918478000	0.000000000
H	-2.112683000	-1.691187000	0.000000000

$(PhOH)_2$.

C	-1.649512000	-0.578063000	1.061469000
C	0.583884000	-0.116528000	0.333267000
C	0.163060000	1.109045000	-0.163821000
C	-1.163279000	1.481084000	-0.041371000
C	-2.077519000	0.643930000	0.569969000
C	-0.328641000	-0.960895000	0.947994000
O	1.866418000	-0.533323000	0.246213000
H	0.014982000	-1.908466000	1.327034000
H	-2.351606000	-1.241541000	1.539258000
H	0.873397000	1.761312000	-0.646022000
H	-3.109219000	0.937542000	0.661791000
H	-1.480498000	2.434739000	-0.430502000
C	4.576153000	4.498713000	2.018150000
C	3.194538000	2.649780000	1.345587000
C	5.279472000	4.400728000	0.832508000
C	4.947582000	3.434988000	-0.099766000
C	3.906318000	2.565327000	0.163393000
C	3.535909000	3.620342000	2.266933000
O	3.538493000	1.596281000	-0.730010000
H	4.836239000	5.251378000	2.742370000
H	2.388108000	1.960865000	1.533053000
H	4.118174000	1.611683000	-1.490261000
H	6.092010000	5.077157000	0.626554000
H	5.495369000	3.357582000	-1.026699000
H	2.982170000	3.687123000	3.188390000
H	2.411954000	0.145580000	-0.164090000

$(PhOH)_3$, conf. 1.

C	-1.869537000	-0.543780000	0.412971000

C	0.502015000	-0.307657000	0.244027000
C	0.435240000	0.899342000	0.918677000
C	-0.792654000	1.375642000	1.339407000
C	-1.949496000	0.660877000	1.089634000
C	-0.649237000	-1.032811000	-0.010811000
O	1.680916000	-0.815467000	-0.211963000
H	-0.572735000	-1.972041000	-0.532409000
H	-2.763426000	-1.111577000	0.214611000
H	1.334087000	1.464291000	1.107512000
H	-2.902815000	1.037150000	1.419007000
H	-0.839935000	2.315784000	1.863302000
C	3.649801000	4.839664000	0.726572000
C	4.141993000	2.549788000	1.259819000
C	3.359226000	4.466604000	-0.572688000
C	3.453741000	3.144038000	-0.964287000
C	3.847471000	2.188246000	-0.043581000
C	4.040609000	3.874323000	1.638083000
O	3.937743000	0.868275000	-0.367688000
H	3.573603000	5.870879000	1.026014000
H	4.451106000	1.789934000	1.957755000
H	3.763420000	0.737056000	-1.310069000
H	3.051752000	5.206948000	-1.292349000
H	3.216822000	2.855475000	-1.975983000
H	4.272176000	4.152653000	2.652814000
H	2.421827000	-0.261080000	0.069752000
H	0.044773000	3.219268000	-5.780451000
C	0.584389000	2.537101000	-5.146145000
C	-0.061345000	1.887616000	-4.110350000
H	-1.108932000	2.063435000	-3.930885000
C	0.625283000	1.011035000	-3.290831000
H	0.120239000	0.513654000	-2.478212000
H	2.137776000	-0.482820000	-2.070453000
O	2.703184000	-0.054609000	-2.728124000
C	1.971908000	0.782327000	-3.515770000
C	2.628877000	1.425515000	-4.550659000
H	3.675518000	1.227963000	-4.710654000
C	1.931146000	2.300652000	-5.360082000
H	2.445078000	2.798477000	-6.165571000

$(PhOH)_3$, conf. 2.

C	-3.874806000	-1.949884000	-3.415482000
C	-1.653615000	-1.654455000	-2.600020000
C	-2.012659000	-0.453285000	-2.019271000
C	-3.315876000	-0.009159000	-2.138955000
C	-4.252034000	-0.751963000	-2.834558000
C	-2.576462000	-2.407536000	-3.301108000
O	-0.375984000	-2.139177000	-2.515374000
H	-2.271739000	-3.340468000	-3.745223000
H	-4.595335000	-2.536208000	-3.960933000
H	-1.275165000	0.137501000	-1.498098000
H	-5.265518000	-0.400430000	-2.925534000
H	-3.593777000	0.929525000	-1.689364000
C	-0.330629000	2.733428000	-2.366173000
C	1.277286000	1.295461000	-1.306644000
C	-0.185125000	2.015987000	-3.539188000
C	0.683327000	0.943274000	-3.608449000
C	1.418131000	0.582719000	-2.488265000
C	0.406659000	2.366887000	-1.253245000
O	2.262198000	-0.477046000	-2.483135000
H	-1.008725000	3.568221000	-2.319245000
H	1.858872000	1.002369000	-0.448827000
H	2.236711000	-0.931532000	-3.334874000
H	-0.757600000	2.285540000	-4.411001000
H	0.790741000	0.380011000	-4.521164000
H	0.305531000	2.918876000	-0.333370000
H	0.158975000	-1.565445000	-1.962058000
H	3.201297000	-6.583061000	-6.680361000
C	2.828439000	-5.669280000	-6.250481000
C	1.774462000	-5.697521000	-5.356196000
H	1.321778000	-6.636583000	-5.084043000
C	1.291952000	-4.527402000	-4.800287000
H	0.475133000	-4.547953000	-4.096142000
H	0.687014000	-2.292297000	-4.023227000
O	1.428033000	-2.140336000	-4.628469000
C	1.870830000	-3.317782000	-5.145939000
C	2.926614000	-3.278066000	-6.039476000
H	3.361794000	-2.327019000	-6.296362000
C	3.399911000	-4.454324000	-6.586407000
H	4.222036000	-4.419533000	-7.281891000

$(PhOH)_3$, conf. 3.

C	3.317371000	0.266333000	0.554858000
C	1.807213000	-1.294045000	-0.434001000
C	1.310832000	-1.622212000	0.812249000

C	1.830232000	-0.998552000	1.930439000
C	2.834385000	-0.055890000	1.809170000
C	2.808069000	-0.352289000	-0.570538000
O	1.254812000	-1.920238000	-1.524184000
H	3.161735000	-0.079775000	-1.552915000
H	4.081882000	1.016522000	0.444395000
H	0.525507000	-2.353428000	0.899501000
H	3.226850000	0.435380000	2.682718000
H	1.440386000	-1.251067000	2.902358000
C	1.303924000	3.618449000	1.043695000
C	1.454523000	3.011133000	-1.276255000
C	0.341534000	2.642391000	1.225439000
C	-0.066935000	1.848072000	0.171356000
C	0.496058000	2.028445000	-1.083648000
C	1.853126000	3.798140000	-0.214476000
O	0.163149000	1.255516000	-2.141992000
H	1.619395000	4.233529000	1.869157000
H	1.876913000	3.140514000	-2.258642000
H	-0.508064000	0.612069000	-1.881918000
H	-0.093239000	2.487391000	2.199245000
H	-0.801415000	1.072654000	0.322626000
H	2.599669000	4.559380000	-0.372429000
H	1.628415000	-1.546269000	-2.322876000
H	-4.903374000	-2.439245000	2.068653000
C	-4.168482000	-2.150923000	1.336833000
C	-3.178486000	-3.037547000	0.955185000
H	-3.140559000	-4.023941000	1.386918000
C	-2.233858000	-2.673626000	0.013765000
H	-1.471196000	-3.369927000	-0.297783000
H	-0.607605000	-1.589983000	-1.504727000
O	-1.376104000	-1.004126000	-1.477587000
C	-2.278055000	-1.407421000	-0.545355000
C	-3.267083000	-0.513733000	-0.172873000
H	-3.288505000	0.464575000	-0.623223000
C	-4.207627000	-0.891145000	0.765401000
H	-4.975829000	-0.192547000	1.052589000

$(PhOH)_3$, conf. 4.

C	2.869205000	-2.761666000	-0.864112000
C	1.723270000	-0.674411000	-1.009618000
C	2.669906000	-0.085801000	-0.190922000
C	3.718049000	-0.848632000	0.288336000
C	3.822208000	-2.187654000	-0.041605000
C	1.818651000	-2.009980000	-1.354220000
O	0.666426000	0.039045000	-1.500189000
H	1.080607000	-2.440405000	-2.010094000
H	2.944729000	-3.802106000	-1.133107000
H	2.581976000	0.958224000	0.068383000
H	4.639467000	-2.777772000	0.336100000
H	4.455144000	-0.390144000	0.926277000
C	1.977478000	3.970693000	-0.268026000
C	1.021820000	2.956059000	1.693106000
C	0.886763000	3.548527000	-1.005650000
C	-0.137299000	2.832709000	-0.405943000
C	-0.063035000	2.515391000	0.947276000
C	2.027983000	3.679693000	1.085745000
O	-1.013995000	1.801557000	1.573885000
H	2.768966000	4.529503000	-0.736653000
H	1.060556000	2.712922000	2.741225000
H	-1.545654000	1.319722000	0.925692000
H	0.818197000	3.787960000	-2.054326000
H	-1.004746000	2.534679000	-0.974004000
H	2.865933000	4.013765000	1.675261000
H	0.780012000	0.969873000	-1.277058000
H	-4.502018000	-4.222721000	0.990034000
C	-3.967173000	-3.339000000	0.687002000
C	-2.655816000	-3.429064000	0.258635000
H	-2.161045000	-4.385681000	0.229326000
C	-1.963508000	-2.297234000	-0.129037000
H	-0.935534000	-2.364518000	-0.447558000
H	-1.084781000	-0.127082000	-0.839820000
O	-1.952626000	0.078176000	-0.461283000
C	-2.594155000	-1.065723000	-0.092367000
C	-3.905422000	-0.963150000	0.336404000
H	-4.378182000	0.004343000	0.356638000
C	-4.584522000	-2.100969000	0.725422000
H	-5.605588000	-2.017882000	1.058927000

1.1.2 XYZ coordinates (SCS-MP2, Å)

PhOH, aug-cc-pVQZ.

C	1.212927000	0.252046000	0.000000000

C	1.216944000	-1.138224000	-0.000001000
C	-0.000599000	0.935990000	0.000000000
C	-1.202728000	0.232742000	0.000000000
C	-1.187832000	-1.160547000	0.000000000
C	0.019397000	-1.852160000	0.000000000
O	0.052782000	2.304623000	0.000000000
H	-0.842892000	2.651108000	-0.000001000
H	2.133823000	0.817660000	0.000001000
H	2.161214000	-1.665217000	0.000001000
H	-2.144186000	0.768747000	0.000001000
H	0.028241000	-2.932628000	-0.000001000
H	-2.124510000	-1.700854000	0.000001000

$(PhOH)_2$, aug-cc-pVQZ.

C	-1.571144000	-0.202422000	1.085774000
C	0.700755000	-0.104932000	0.287222000
C	0.479639000	1.193121000	-0.175033000
C	-0.768443000	1.787229000	0.001226000
C	-1.799071000	1.095822000	0.630900000
C	-0.328808000	-0.803637000	0.917585000
O	1.900192000	-0.740912000	0.154935000
H	-0.139126000	-1.808590000	1.267833000
H	-2.363828000	-0.751477000	1.575721000
H	1.277825000	1.733609000	-0.666333000
H	-2.765647000	1.560146000	0.765256000
H	-0.929573000	2.794833000	-0.357586000
C	4.002117000	4.454384000	1.931724000
C	3.459481000	2.135429000	1.505581000
C	4.545200000	4.560460000	0.655168000
C	4.546790000	3.462034000	-0.202604000
C	4.004327000	2.257280000	0.230943000
C	3.460290000	3.240176000	2.350439000
O	3.968820000	1.139160000	-0.577599000
H	4.000384000	5.308229000	2.593853000
H	3.043451000	1.188244000	1.819571000
H	4.403201000	1.335010000	-1.412063000
H	4.968272000	5.496819000	0.318942000
H	4.966092000	3.544765000	-1.198017000
H	3.034241000	3.148555000	3.339781000
H	2.548517000	-0.136970000	-0.231833000

PhOH, aug-cc-pVTZ.

C	1.214864000	0.252661000	0.000000000
C	1.218816000	-1.139776000	0.000000000
C	-0.000616000	0.937679000	0.000000000
C	-1.204714000	0.233481000	0.000000000
C	-1.189789000	-1.162063000	0.000000000
C	0.019334000	-1.855151000	0.000000000
O	0.054454000	2.308897000	0.000000000
H	-0.844232000	2.655087000	0.000000000
H	2.136355000	0.819371000	0.000000000
H	2.164369000	-1.666857000	0.000000000
H	-2.147329000	0.769694000	0.000000000
H	0.028547000	-2.936730000	0.000000000
H	-2.127476000	-1.703008000	0.000000000

$(PhOH)_2$, aug-cc-pVTZ.

C	-1.495382000	-0.069336000	1.130315000
C	0.761014000	-0.083462000	0.272844000
C	0.584913000	1.218115000	-0.204551000
C	-0.632006000	1.871280000	-0.004207000
C	-1.677732000	1.233734000	0.661772000
C	-0.283688000	-0.728862000	0.938771000
O	1.929195000	-0.776227000	0.117860000
H	-0.129201000	-1.737784000	1.298431000
H	-2.299406000	-0.576723000	1.648554000
H	1.393497000	1.716674000	-0.724171000
H	-2.620242000	1.742596000	0.814085000
H	-0.757504000	2.881423000	-0.373734000
C	3.804699000	4.388290000	1.921175000
C	3.484987000	2.020181000	1.517710000
C	4.319331000	4.536668000	0.634499000
C	4.416844000	3.434670000	-0.217224000
C	3.998895000	2.184918000	0.232795000
C	3.388210000	3.128302000	2.355970000
O	4.055821000	1.059759000	-0.569989000
H	3.727306000	5.244186000	2.578065000
H	3.164397000	1.038783000	1.841888000
H	4.466364000	1.291020000	-1.410599000
H	4.644955000	5.508155000	0.285654000

H	4.813321000	3.549942000	-1.220077000
H	2.984274000	3.003413000	3.352216000
H	2.601094000	-0.193351000	-0.266668000

$(PhOH)_3$, conf. 1, aug-cc-pVTZ.

C	-1.743788000	-0.348325000	0.262411000
C	0.664768000	-0.272984000	0.242257000
C	0.638678000	1.005920000	0.799281000
C	-0.590854000	1.603855000	1.080157000
C	-1.784755000	0.934953000	0.811491000
C	-0.522486000	-0.957073000	-0.021237000
O	1.837655000	-0.904370000	-0.108452000
H	-0.474761000	-1.951297000	-0.447000000
H	-2.663334000	-0.880899000	0.055293000
H	1.561908000	1.536326000	0.999443000
H	-2.734742000	1.404873000	1.028930000
H	-0.606762000	2.598779000	1.506951000
C	3.190561000	4.653522000	0.678387000
C	4.128022000	2.471473000	1.154097000
C	2.924563000	4.246061000	-0.628617000
C	3.250658000	2.956922000	-1.052506000
C	3.844442000	2.071371000	-0.152222000
C	3.797471000	3.761521000	1.565180000
O	4.136997000	0.767142000	-0.481874000
H	2.933130000	5.653423000	1.001680000
H	4.596314000	1.767859000	1.830615000
H	3.891589000	0.598385000	-1.408904000
H	2.457311000	4.927641000	-1.328257000
H	3.030534000	2.643849000	-2.066006000
H	4.016664000	4.068060000	2.580119000
H	2.599304000	-0.341786000	0.116528000
H	0.114905000	3.480703000	-5.347687000
C	0.635508000	2.700019000	-4.809080000
C	0.015779000	2.045825000	-3.744604000
H	-0.990067000	2.316453000	-3.449129000
C	0.682081000	1.040241000	-3.042433000
H	0.199719000	0.545226000	-2.207957000
H	2.182077000	-0.637224000	-2.018479000
O	2.715593000	-0.254894000	-2.737840000
C	1.980652000	0.691661000	-3.415621000
C	2.607817000	1.332049000	-4.484922000
H	3.613480000	1.038966000	-4.758152000
C	1.932477000	2.335935000	-5.176862000
H	2.422503000	2.832014000	-6.005042000

$(PhOH)_3$, conf. 2, aug-cc-pVTZ.

C	-3.889346000	-1.816173000	-3.488838000
C	-1.655991000	-1.696173000	-2.603931000
C	-1.973328000	-0.524314000	-1.920971000
C	-3.262788000	-0.002699000	-2.028865000
C	-4.224981000	-0.644076000	-2.808477000
C	-2.605456000	-2.348949000	-3.387517000
O	-0.396024000	-2.263862000	-2.544325000
H	-2.332930000	-3.259425000	-3.906059000
H	-4.627375000	-2.321376000	-4.098617000
H	-1.221598000	-0.013585000	-1.330555000
H	-5.223263000	-0.234848000	-2.888461000
H	-3.506993000	0.911206000	-1.502324000
C	-0.324998000	2.778334000	-2.388203000
C	1.228414000	1.267528000	-1.304437000
C	-0.199796000	2.047457000	-3.569305000
C	0.630907000	0.928007000	-3.628684000
C	1.339565000	0.536913000	-2.489764000
C	0.396863000	2.385418000	-1.259035000
O	2.131138000	-0.583415000	-2.461776000
H	-0.971548000	3.644973000	-2.348416000
H	1.794322000	0.951007000	-0.437512000
H	2.116690000	-1.008532000	-3.336965000
H	-0.754812000	2.340834000	-4.451415000
H	0.721340000	0.354522000	-4.543200000
H	0.315273000	2.949890000	-0.338552000
H	0.186859000	-1.711533000	-2.003014000
H	3.366295000	-6.589673000	-6.593220000
C	2.938970000	-5.678008000	-6.197739000
C	1.916302000	-5.733818000	-5.251479000
H	1.545986000	-6.690850000	-4.906310000
C	1.361965000	-4.560949000	-4.737642000
H	0.572976000	-4.599649000	-3.995800000
H	0.602181000	-2.331286000	-4.078962000
O	1.326312000	-2.141223000	-4.705370000
C	1.838346000	-3.326350000	-5.179937000
C	2.860738000	-3.257717000	-6.125571000
H	3.211456000	-2.288426000	-6.455413000
C	3.408443000	-4.435954000	-6.629200000

H	4.202562000	-4.381174000	-7.362986000

$(PhOH)_3$, conf. 3, aug-cc-pVTZ.

C	3.276831000	0.345611000	0.773276000
C	1.782253000	-1.155440000	-0.369320000
C	1.329273000	-1.651699000	0.849575000
C	1.857469000	-1.139214000	2.033742000
C	2.827292000	-0.137660000	2.001461000
C	2.760730000	-0.165155000	-0.415935000
O	1.221129000	-1.677398000	-1.524346000
H	3.100631000	0.217122000	-1.370269000
H	4.021819000	1.128303000	0.734041000
H	0.571540000	-2.422095000	0.867359000
H	3.227214000	0.263022000	2.922476000
H	1.501918000	-1.521872000	2.981009000
C	1.434835000	3.543369000	0.914132000
C	1.488450000	2.950140000	-1.433091000
C	0.448497000	2.580363000	1.124961000
C	-0.020063000	1.800601000	0.068678000
C	0.508145000	1.981561000	-1.212205000
C	1.948642000	3.725205000	-0.370670000
O	0.120356000	1.218282000	-2.280203000
H	1.794831000	4.145691000	1.736793000
H	1.876964000	3.082258000	-2.433964000
H	-0.598002000	0.630139000	-1.994888000
H	0.041681000	2.426103000	2.115652000
H	-0.775523000	1.044439000	0.240273000
H	2.708344000	4.474758000	-0.550382000
H	1.459969000	-1.090384000	-2.253099000
H	-4.872414000	-2.489649000	2.145661000
C	-4.166492000	-2.184877000	1.385952000
C	-3.128386000	-3.035907000	1.009674000
H	-3.026572000	-4.007954000	1.473407000
C	-2.217281000	-2.648338000	0.027481000
H	-1.425612000	-3.317528000	-0.284997000
H	-0.654886000	-1.494013000	-1.513932000
O	-1.474373000	-0.969805000	-1.547547000
C	-2.343108000	-1.395643000	-0.573884000
C	-3.381844000	-0.539849000	-0.208780000
H	-3.468429000	0.422906000	-0.694050000
C	-4.291047000	-0.939822000	0.768269000
H	-5.095217000	-0.273007000	1.048821000

1.2 Calculated vibrational frequencies

Table S1: The selected calculated IR-active harmonic vibrational frequencies (ω, cm^{-1}), located in spectral ranges of interest, and their intensities (I, km·mol^{-1}) using the DFT (PW6B95-D4/ma-def2-QZVP) and the SCS-MP2/aug-cc-pVTZ levels of theory, and the observed band positions for the assigned modes (**exp**, cm^{-1}). Conformation indices are given in parentheses and correspond to the structures in Figure 3 of the main text.

DFT Method

$(PhOH)_2$			$(PhOH)_3$ (1)			$(PhOH)_3$ (2)		$(PhOH)_3$ (3)		$(PhOH)_3$ (4)		$PhOH\cdot H_2O$ (1)			$PhOH\cdot H_2O$ (2)	
ω	I	**exp**	ω	I	**exp**	ω	I	ω	I	ω	I	ω	I	**exp**	ω	I
685	95	**605**	578	244	**557**	519	35	445	28	525	27	722	124	**642**	531	80
713	35		586	240		536	106	610	115	600	145	778	43		542	62
721	23		783	52		620	162	735	192	733	82	3704	663		776	53
777	54		783	44		779	73	780	90	783	110					
779	37		828	121	**781**	783	73	3657	330	3618	438					
3744	597		3670	826		798	25	3695	908	3678	1002					
			3672	817		3627	579	3859	68	3723	540					
						3694	978									
						3824	148									

SCS-MP2 Method

$(PhOH)_2$			$(PhOH)_3$ (1)			$(PhOH)_3$ (2)		$(PhOH)_3$ (3)		$(PhOH)_3$ (4)		$PhOH\cdot H_2O$ (1)			$PhOH\cdot H_2O$ (2)	
ω	I	**exp**	ω	I	**exp**	ω	I	ω	I	ω	I	ω	I	**exp**	ω	I
636	70	**605**	575	208	**557**	484	98	444	43	515	27	711	117	**642**	511	104
653	54		577	209		519	59	593	122	536	30	766	51		534	20
660	33		765	84		611	171	746	235	584	151	3697	600		763	68
759	100		765	82		810	33	758	47	730	90					
760	34		813	96	**781**	3571	522	3603	277	3588	320					
3709	459		3653	597		3644	945	3646	888	3643	813					
			3656	581		3754	171	3799	66	3677	513					

Table S2: The calculated harmonic vibrational frequencies (ω, cm^{-1}) and intensities (I, km/mol) of phenol (PhOH) and phenol dimer $(PhOH)_2$ using the SCS-MP2 method on aug-cc-pVTZ (ATZ) and aug-cc-pVQZ (AQZ) basis sets.

PhOH, AQZ		PhOH, ATZ		$(PhOH)_2$, AQZ		$(PhOH)_2$, ATZ	
ω, cm^{-1}	I, km/mol	ω, cm^{-1}	I, km/mol	ω, cm^{-1}	I, km/mol	ω, cm^{-1}	I, km/mol
228.4	1.3	220.4	1.1	10.1	0.1	10.3	0.2
326.3	96.5	323.8	94.9	23.6	0.5	24.3	0.1
403.1	9.5	399.9	1.6	33.8	0.1	31.1	0.7
413.9	0.7	401.8	9.4	61.4	0.7	63.2	0.5
511.0	12.0	452.1	2.6	74.0	0.6	80.3	0.6
530.1	1.7	526.8	1.6	114.0	2.4	116.0	2.2

Table S2 continued from previous page

PhOH, AQZ		PhOH, ATZ		$(PhOH)_2$, AQZ		$(PhOH)_2$, ATZ	
ω, cm^{-1}	I, km/mol	ω, cm^{-1}	I, km/mol	ω, cm^{-1}	I, km/mol	ω, cm^{-1}	I, km/mol
624.2	0.3	556.0	13.9	233.6	0.2	232.3	0.2
674.9	15.7	620.8	0.3	239.0	0.6	239.8	0.5
762.8	67.6	754.5	79.4	322.5	90.2	326.9	89.3
826.0	21.3	822.6	21.7	409.3	3.8	406.0	3.4
834.5	0.0	826.4	0.0	414.7	2.2	411.8	0.7
901.1	4.6	870.8	3.1	418.7	0.1	412.8	1.3
971.4	0.0	914.4	1.0	422.8	0.7	419.8	1.2
983.6	0.0	944.3	0.0	512.3	14.2	506.3	3.0
1019.8	1.5	1016.6	1.0	516.6	4.1	508.0	13.0
1045.9	5.3	1043.5	5.4	530.0	1.2	527.0	1.0
1095.0	11.7	1092.3	11.3	532.1	2.5	528.2	2.4
1174.6	12.6	1168.9	8.5	623.6	1.6	619.9	1.7
1193.6	4.5	1188.4	8.7	623.8	2.2	620.5	4.5
1202.9	135.4	1200.8	135.0	638.0	119.1	632.3	87.8
1285.8	79.2	1282.1	76.0	692.3	12.7	654.2	35.9
1370.7	3.2	1361.7	13.4	694.7	41.5	659.2	32.8
1379.5	27.0	1381.5	17.3	765.0	84.6	759.5	105.1
1506.1	21.0	1499.4	21.6	768.7	27.6	760.9	28.5
1537.7	52.1	1531.0	52.4	822.6	24.4	819.2	25.1
1647.9	44.8	1645.0	45.6	828.6	18.4	825.7	17.8
1658.8	32.3	1655.5	31.2	837.6	0.9	831.1	0.2
3188.2	9.8	3181.7	9.4	840.4	2.3	833.9	2.3
3205.0	0.1	3197.3	0.1	904.9	3.5	891.3	8.1
3213.6	12.3	3207.3	13.4	911.2	7.5	895.4	4.9
3226.6	10.8	3221.3	11.6	975.6	0.1	963.1	0.2
3233.3	5.0	3229.4	5.3	982.0	0.0	964.0	0.1
3848.5	67.5	3829.5	65.0	994.0	0.3	965.9	0.0
				1004.4	0.2	974.8	0.3
				1019.2	2.2	1016.3	1.4
				1021.1	0.6	1017.1	0.3
				1045.2	4.9	1043.2	5.1
				1047.3	5.5	1044.4	5.0
				1096.2	2.6	1092.9	5.1
				1101.0	14.2	1095.7	10.9
				1173.6	3.2	1168.5	1.3

Table S2 continued from previous page

PhOH, AQZ		PhOH, ATZ		$(PhOH)_2$, AQZ		$(PhOH)_2$, ATZ	
ω, cm^{-1}	I, km/mol	ω, cm^{-1}	I, km/mol	ω, cm^{-1}	I, km/mol	ω, cm^{-1}	I, km/mol
				1177.3	6.9	1171.8	5.5
				1192.0	6.8	1186.7	9.7
				1196.8	0.5	1191.5	1.4
				1208.6	134.0	1205.3	134.5
				1243.3	122.9	1243.9	112.3
				1269.3	72.3	1265.4	65.5
				1293.9	64.0	1290.4	59.0
				1372.5	5.8	1363.9	14.6
				1373.0	0.2	1367.4	6.0
				1380.4	24.2	1382.1	14.1
				1396.0	32.8	1396.5	29.5
				1507.2	29.1	1500.7	26.0
				1509.6	16.6	1502.3	19.0
				1535.2	45.5	1527.8	39.0
				1539.4	51.9	1533.2	54.9
				1643.9	36.8	1640.9	32.7
				1648.8	53.5	1645.2	56.0
				1656.9	9.6	1654.0	5.6
				1659.7	43.8	1655.7	44.0
				3189.9	7.8	3183.2	7.5
				3198.4	2.7	3190.7	2.1
				3204.9	3.7	3198.6	4.5
				3208.7	0.4	3201.4	0.2
				3215.4	4.8	3210.7	8.9
				3216.6	22.0	3212.2	15.4
				3222.5	13.1	3218.1	14.0
				3227.4	6.0	3221.7	5.4
				3231.8	2.4	3226.2	6.4
				3235.4	5.2	3230.2	5.7
				3726.6	469.8	3711.3	439.7
				3842.6	78.0	3823.8	74.2

Table S3: The calculated harmonic vibrational frequencies (ω, cm^{-1}) and intensities (I, km/mol) of the phenol trimer $(PhOH)_3$ clusters using the SCS-MP2 method on aug-cc-pVTZ (ATZ) basis set.

$(PhOH)_3$, conf. 1		$(PhOH)_3$, conf. 2		$(PhOH)_3$, conf. 3	
ω, cm^{-1}	I, km/mol	ω, cm^{-1}	I, km/mol	ω, cm^{-1}	I, km/mol
11.3	0.2	8.9	0.5	0.0	0.0
18.5	0.2	18.2	0.1	14.2	3.2
29.5	0.1	21.0	0.2	16.3	0.3
33.6	0.0	30.9	0.7	35.4	0.3
37.2	0.1	41.8	0.2	48.8	1.7
49.0	0.0	49.3	1.1	56.3	4.3
64.6	0.7	58.3	0.1	58.5	0.1
64.9	0.6	61.6	0.9	67.9	0.3
80.3	0.2	78.9	0.1	77.0	0.5
117.3	12.9	99.6	5.6	91.3	3.2
121.6	12.6	127.5	4.3	124.3	2.5
146.1	0.1	143.6	5.0	138.8	4.3
243.0	3.3	238.4	1.1	240.5	7.6
244.8	3.6	243.5	3.1	243.7	1.7
255.4	0.2	251.5	2.7	248.8	6.3
410.9	0.6	409.1	4.5	368.6	129.1
412.1	0.6	412.2	0.7	411.4	0.8
414.5	0.8	413.1	0.1	413.8	6.9
419.6	1.2	414.4	1.1	414.5	1.2
420.3	1.2	418.0	1.2	417.3	1.1
430.2	9.2	436.7	5.2	426.2	1.7
511.0	0.5	484.7	100.2	444.3	49.8
511.4	0.6	501.9	7.8	506.1	13.8
513.1	1.5	507.4	5.9	513.0	5.1
527.9	5.3	519.6	57.7	517.9	6.4
528.3	3.0	527.5	3.5	528.5	3.6
531.3	6.5	531.5	24.0	529.9	0.8
575.0	209.9	534.3	2.3	535.5	6.9
581.2	210.0	615.9	137.6	598.3	145.0
619.4	1.0	619.6	2.5	621.2	1.3
620.3	0.5	620.6	15.5	622.4	1.1
620.4	0.8	621.0	8.1	623.5	3.1

Table S3 continued from previous page

$(PhOH)_3$, conf. 1		$(PhOH)_3$, conf. 2		$(PhOH)_3$, conf. 3	
ω, cm^{-1}	I, km/mol	ω, cm^{-1}	I, km/mol	ω, cm^{-1}	I, km/mol
676.2	2.8	633.5	20.7	678.2	21.9
679.2	27.0	669.5	12.9	683.3	5.7
679.4	25.5	677.7	29.3	686.5	15.5
765.0	87.0	759.0	32.0	740.1	187.4
765.2	82.1	761.3	90.7	758.5	94.5
765.6	19.9	765.9	72.3	765.5	12.2
814.6	92.2	806.5	31.2	765.7	60.1
821.3	9.7	822.7	1.9	818.9	17.4
821.9	11.2	823.6	57.0	820.0	2.8
835.0	3.3	830.1	2.5	826.0	19.5
838.1	0.9	835.2	1.0	831.5	16.3
838.3	0.9	843.3	0.8	835.0	0.4
866.5	41.6	851.3	3.7	842.2	9.0
903.3	1.5	890.7	3.5	885.9	11.6
904.1	7.1	901.0	8.8	894.7	3.8
905.1	7.7	905.5	6.2	900.4	9.5
971.8	0.2	956.0	0.1	949.8	1.0
972.6	0.2	967.3	0.0	953.8	0.2
973.7	0.0	969.1	0.2	957.9	0.0
977.8	0.2	971.2	0.0	960.1	0.1
978.7	0.2	976.5	0.1	960.9	0.5
979.4	0.2	978.0	0.4	964.4	0.1
1015.8	0.9	1016.0	1.5	1016.4	0.8
1016.6	0.7	1016.9	0.8	1018.0	0.1
1016.9	0.6	1017.1	0.7	1019.0	0.3
1042.6	2.9	1042.8	3.4	1043.3	3.8
1042.8	3.1	1043.0	14.5	1044.0	5.2
1043.4	4.9	1043.5	1.1	1045.4	2.1
1095.6	7.5	1093.7	3.2	1095.7	8.5
1095.8	7.6	1094.6	9.3	1095.9	3.8
1097.1	0.1	1095.5	6.7	1097.6	11.6
1170.1	0.3	1169.8	1.9	1172.7	0.8
1170.1	0.4	1170.3	1.7	1175.2	3.2
1170.7	1.7	1170.6	3.3	1176.3	5.3
1191.2	3.7	1187.8	5.9	1189.2	5.5

Table S3 continued from previous page

$(PhOH)_3$, conf. 1		$(PhOH)_3$, conf. 2		$(PhOH)_3$, conf. 3	
ω, cm^{-1}	I, km/mol	ω, cm^{-1}	I, km/mol	ω, cm^{-1}	I, km/mol
1191.3	5.2	1189.6	5.5	1189.5	4.3
1191.3	16.7	1190.6	7.6	1192.5	8.4
1226.5	80.0	1210.4	98.0	1213.0	157.9
1229.8	84.4	1252.6	147.9	1247.5	160.7
1274.9	113.9	1256.8	376.4	1262.8	21.5
1279.0	19.6	1270.8	78.6	1283.7	104.0
1280.3	15.7	1286.5	5.6	1293.0	25.4
1285.0	44.7	1291.1	13.6	1297.1	39.1
1368.7	2.0	1365.1	15.2	1368.6	9.0
1369.0	1.3	1368.8	2.7	1371.4	3.5
1371.4	14.2	1370.5	5.4	1374.1	7.3
1390.5	18.2	1383.6	17.0	1390.6	12.4
1392.1	19.5	1399.8	9.9	1404.3	44.3
1407.3	55.9	1411.4	130.3	1414.9	43.9
1500.1	25.2	1498.3	13.9	1507.2	7.3
1500.7	28.1	1503.7	26.7	1508.1	57.1
1506.7	38.8	1507.3	152.9	1511.8	48.0
1530.1	16.7	1527.9	35.6	1531.8	33.5
1530.3	19.3	1532.4	59.3	1536.4	47.2
1532.6	67.1	1533.0	17.9	1539.4	33.0
1639.1	21.3	1638.3	26.5	1646.6	23.9
1639.3	24.1	1640.8	160.7	1648.9	55.1
1645.8	66.0	1645.3	21.1	1654.2	36.4
1652.4	12.6	1652.3	11.7	1661.7	12.0
1652.9	13.4	1653.0	19.1	1662.6	23.9
1654.4	26.8	1657.2	15.7	1665.3	26.5
3192.9	1.8	3191.9	5.7	3198.4	2.9
3193.8	1.1	3192.0	1.6	3200.4	7.1
3194.0	1.3	3193.6	1.1	3202.2	3.4
3200.1	8.2	3198.8	2.0	3204.8	5.1
3200.8	11.1	3199.5	1.7	3208.4	0.3
3200.9	6.2	3200.9	4.6	3209.1	1.2
3209.8	1.7	3207.9	5.5	3214.6	10.4
3210.2	0.7	3209.3	8.9	3216.7	12.3
3210.2	4.1	3211.9	3.2	3219.4	26.6

Table S3 continued from previous page

(PhOH)$_3$, conf. 1		(PhOH)$_3$, conf. 2		(PhOH)$_3$, conf. 3	
ω, cm^{-1}	I, km/mol	ω, cm^{-1}	I, km/mol	ω, cm^{-1}	I, km/mol
3216.7	6.4	3215.8	12.1	3221.2	2.1
3216.8	7.5	3217.1	7.6	3224.1	14.9
3217.1	6.5	3218.4	12.7	3229.8	19.6
3226.5	9.6	3226.2	7.7	3231.1	2.8
3226.6	10.1	3227.1	9.2	3235.1	7.5
3226.8	9.3	3227.9	9.9	3238.2	3.0
3608.8	24.6	3568.7	575.1	3624.6	320.3
3649.3	619.4	3647.0	901.7	3673.7	853.8
3657.4	566.2	3751.4	169.7	3798.5	73.4

Table S4: The calculated harmonic vibrational frequencies (ω, cm^{-1}) and intensities (I, km/mol) of phenol (PhOH) and phenol dimer (PhOH)$_2$ using the PW6B95-D4 method on ma-def2-QZVP basis set.

PhOH		(PhOH)$_2$	
ω, cm^{-1}	I, km/mol	ω, cm^{-1}	I, km/mol
230.4	0.9	15.6	0.2
360.3	99.2	33.7	0.0
406.0	9.9	40.6	0.6
422.3	0.3	83.3	0.7
519.3	12.1	109.6	0.6
535.1	1.6	117.3	3.0
630.2	0.4	233.7	1.8
710.7	28.9	243.6	0.3
776.2	52.6	335.5	86.2
838.1	0.0	415.4	4.5
840.5	20.4	421.8	0.3
911.7	6.6	428.6	1.3
990.0	0.0	430.6	1.1
1011.6	0.2	518.7	11.5
1023.5	1.2	525.9	8.2
1059.6	4.7	535.3	1.7
1105.1	12.7	539.7	2.5
1180.2	14.3	629.4	1.5
1196.2	13.0	631.0	0.1

Table S4 continued from previous page

PhOH		$(PhOH)_2$	
ω, cm^{-1}	I, km/mol	ω, cm^{-1}	I, km/mol
1202.6	124.8	685.2	94.9
1303.7	94.3	713.0	35.2
1370.8	2.7	721.2	22.5
1381.1	27.5	776.8	54.4
1520.4	21.7	779.3	37.2
1550.9	63.3	836.1	29.1
1669.0	47.3	842.0	7.0
1679.0	42.4	843.5	13.1
3184.1	13.8	856.7	1.0
3204.7	0.2	918.9	8.1
3213.1	14.9	925.2	4.2
3226.5	14.9	994.6	0.2
3232.9	5.4	998.0	0.0
3880.9	64.7	1012.3	0.4
		1018.9	0.2
		1022.4	1.9
		1024.9	0.4
		1059.0	6.2
		1065.4	4.5
		1106.6	6.0
		1120.2	16.9
		1180.2	2.6
		1184.5	4.4
		1196.2	8.0
		1204.7	52.2
		1221.9	98.4
		1250.7	103.1
		1286.3	118.5
		1313.0	92.8
		1370.9	5.3
		1373.9	7.4
		1388.5	11.0
		1398.8	34.3
		1522.1	30.4
		1524.6	11.5

Table S4 continued from previous page

PhOH		$(PhOH)_2$	
ω, cm^{-1}	I, km/mol	ω, cm^{-1}	I, km/mol
		1550.4	86.9
		1555.2	48.4
		1663.8	47.3
		1672.1	66.7
		1678.4	39.7
		1681.7	31.1
		3187.0	11.1
		3197.3	4.2
		3203.9	3.8
		3209.6	0.1
		3213.4	15.0
		3218.0	12.7
		3223.5	17.2
		3230.3	8.8
		3231.7	12.5
		3240.1	1.0
		3744.0	596.5
		3880.1	76.8

Table S5: The calculated harmonic vibrational frequencies (ω, cm^{-1}) and intensities (I, km/mol) of the phenol trimer $(PhOH)_3$ clusters using the PW6B95-D4 method on ma-def2-QZVP basis set.

$(PhOH)_3$, conf. 1		$(PhOH)_3$, conf. 2		$(PhOH)_3$, conf. 3		$(PhOH)_4$, conf. 3	
ω, cm^{-1}	I, km/mol	ω, cm^{-1}	I, km/mol	ω, cm^{-1}	I, km/mol	ω, cm^{-1}	I, km/mol
20.9	0.1	16.7	0.1	25.2	0.0	17.4	0.3
20.9	0.1	20.1	0.5	34.1	0.1	21.7	0.1
31.4	0.1	26.0	0.2	46.4	1.6	33.3	0.2
39.8	0.1	41.8	0.6	52.4	0.9	33.9	0.2
41.4	0.1	51.2	0.2	70.2	0.2	46.1	0.2
43.4	0.1	67.2	0.2	74.4	0.2	58.9	0.2
59.3	1.4	71.0	1.2	82.2	2.0	70.2	0.3
61.8	0.9	82.6	0.4	104.0	1.9	76.3	0.5
84.9	0.3	101.6	2.6	117.6	3.3	96.8	0.1
116.7	14.0	115.7	1.1	129.9	3.8	119.1	2.7
119.5	13.7	127.3	3.3	155.6	3.1	130.4	2.9
137.3	0.2	157.8	5.7	159.5	2.3	140.8	8.8
245.7	3.0	243.9	0.2	246.4	1.1	242.9	0.1
246.9	3.1	248.7	2.2	253.4	4.4	253.0	1.2
255.8	0.1	263.4	2.0	266.0	13.2	256.9	6.7
421.4	1.4	414.9	3.6	358.8	116.7	420.5	1.9
422.0	1.2	421.4	2.7	415.7	3.9	422.0	0.4
424.4	0.8	423.5	0.7	424.9	1.6	424.1	3.6
424.7	0.6	423.8	0.2	427.8	1.4	426.7	1.0
424.9	0.5	432.6	0.5	432.3	0.6	429.9	1.5
436.0	9.9	440.7	4.8	437.3	0.1	442.6	5.4
525.1	2.2	519.1	34.5	444.9	28.4	519.6	13.0
527.3	3.9	522.3	11.9	516.2	8.8	523.2	0.7
528.1	7.2	525.6	3.0	522.7	5.9	525.3	27.9
536.5	4.9	534.3	12.6	526.6	7.5	532.7	27.7
537.4	3.2	536.0	106.3	533.3	2.9	537.9	8.1
539.9	6.3	539.0	18.4	538.1	0.8	543.5	21.1
578.2	244.4	546.4	25.9	541.7	7.1	548.0	16.6
585.9	240.3	619.6	161.6	610.2	115.5	600.3	145.4
629.5	0.9	629.0	0.9	629.2	1.2	629.4	2.6
630.3	0.4	630.1	1.4	630.4	1.2	630.0	1.6
630.5	0.5	630.9	0.7	631.3	4.3	630.8	1.9

Table S5 continued from previous page

$(PhOH)_3$, conf. 1		$(PhOH)_3$, conf. 2		$(PhOH)_3$, conf. 3		$(PhOH)_3$, conf. 4	
ω, cm^{-1}	I, km/mol	ω, cm^{-1}	I, km/mol	ω, cm^{-1}	I, km/mol	ω, cm^{-1}	I, km/mol
714.3	12.7	715.9	17.9	710.7	46.2	713.2	25.4
717.5	38.9	717.5	27.0	717.6	13.6	718.8	18.0
718.4	43.0	723.7	44.0	720.1	22.7	725.4	32.0
780.6	47.2	773.5	2.3	735.6	192.4	733.9	82.8
782.6	52.0	779.4	72.6	775.2	46.3	779.6	36.9
783.3	43.7	782.7	73.0	779.7	44.1	780.5	53.9
828.4	121.3	797.6	25.1	783.1	29.0	786.4	56.5
838.9	14.6	838.5	37.0	832.4	2.1	838.3	30.5
839.0	15.7	841.2	36.1	833.5	19.9	841.7	32.3
844.6	0.4	844.4	3.2	842.7	21.4	845.4	3.2
847.9	0.4	851.3	2.0	846.0	15.1	847.2	5.4
848.7	0.3	854.6	2.3	847.9	0.6	853.3	0.6
867.6	44.3	861.8	1.0	855.3	9.1	868.2	9.5
921.6	7.7	921.1	5.7	914.3	12.7	918.6	4.7
922.4	8.4	923.7	7.7	919.6	7.3	926.4	6.8
923.8	2.9	937.8	11.1	923.9	9.6	930.1	18.9
997.3	0.1	995.3	0.0	991.2	1.0	993.8	0.1
997.6	0.2	1000.5	0.4	995.9	0.1	998.5	0.4
998.8	0.0	1004.0	0.7	998.4	0.2	1003.7	1.9
1016.7	0.2	1014.6	0.2	1013.2	0.6	1014.6	0.1
1017.6	0.3	1019.0	0.7	1016.9	0.4	1017.7	0.2
1017.9	0.3	1021.3	1.4	1017.5	0.3	1019.0	1.5
1024.0	0.6	1023.9	1.6	1023.4	1.3	1022.4	2.8
1024.7	0.5	1024.8	0.8	1024.4	0.4	1025.1	0.6
1024.9	0.6	1026.5	0.7	1026.9	0.2	1026.1	0.7
1058.8	3.9	1058.9	4.2	1059.7	5.3	1060.2	3.7
1059.0	4.8	1059.4	9.1	1060.3	1.8	1061.3	7.8
1059.5	5.0	1063.6	4.1	1061.9	3.5	1061.8	5.3
1108.6	11.2	1105.8	2.3	1107.9	8.1	1110.7	3.5
1108.8	11.4	1108.8	9.7	1111.9	8.6	1114.0	7.3
1110.3	0.3	1116.8	10.6	1113.4	7.2	1114.8	6.0
1181.9	1.4	1181.6	1.2	1179.7	2.3	1182.1	1.9
1182.1	1.2	1182.1	2.4	1182.6	7.4	1182.3	2.1
1182.5	1.4	1182.9	6.0	1183.1	7.9	1182.8	3.0
1198.2	8.0	1195.4	0.6	1196.8	10.3	1201.7	7.6

Table S5 continued from previous page

(PhOH)$_3$, conf. 1		(PhOH)$_3$, conf. 2		(PhOH)$_3$, conf. 3		(PhOH)$_3$, conf. 4	
ω, cm^{-1}	I, km/mol	ω, cm^{-1}	I, km/mol	ω, cm^{-1}	I, km/mol	ω, cm^{-1}	I, km/mol
1198.3	10.7	1197.2	24.1	1200.1	6.0	1203.1	10.0
1198.7	14.3	1206.2	0.9	1204.6	17.5	1205.2	8.4
1235.7	138.1	1212.3	124.3	1207.5	163.4	1234.8	179.0
1238.3	146.8	1251.7	16.1	1249.0	157.4	1267.5	23.5
1290.7	22.3	1265.8	480.0	1266.2	24.6	1286.5	212.8
1295.3	37.9	1288.9	47.3	1290.8	69.6	1296.0	164.8
1295.7	38.8	1300.0	111.3	1303.4	95.6	1303.9	18.8
1304.8	112.6	1310.2	42.9	1316.8	70.5	1327.9	49.8
1370.3	1.5	1370.5	1.1	1370.4	1.6	1372.6	1.2
1370.9	2.7	1371.0	3.7	1371.6	1.2	1373.3	3.2
1371.1	2.8	1372.9	3.6	1375.9	3.0	1373.6	5.0
1390.4	28.0	1385.6	33.2	1383.8	23.4	1395.6	30.5
1392.4	30.4	1397.8	8.0	1397.7	44.2	1412.8	7.6
1417.6	65.8	1407.9	126.8	1410.1	50.3	1426.6	125.2
1520.6	31.4	1520.9	13.8	1521.5	31.7	1521.1	43.3
1520.9	31.8	1523.8	7.7	1522.9	18.8	1522.9	22.4
1527.2	38.0	1524.5	122.8	1524.6	37.0	1529.6	111.8
1549.8	35.7	1549.5	140.5	1549.3	50.9	1553.2	48.6
1550.5	39.1	1551.4	30.5	1551.1	50.3	1554.3	83.7
1551.9	83.3	1553.0	16.7	1557.2	47.9	1558.0	16.8
1665.9	35.6	1663.7	44.8	1665.7	53.0	1658.3	53.2
1666.2	34.0	1667.3	124.1	1667.2	35.9	1668.1	68.1
1672.7	67.5	1671.9	22.9	1673.4	38.1	1672.4	88.6
1678.7	26.7	1678.1	60.9	1678.6	47.6	1677.3	49.8
1679.0	26.5	1679.9	17.5	1680.6	19.8	1680.3	21.6
1681.0	38.1	1680.7	20.5	1682.2	31.2	1682.4	6.1
3202.1	1.0	3200.1	8.6	3192.9	6.2	3194.7	1.7
3202.3	0.9	3202.6	1.4	3195.6	9.2	3197.7	3.5
3202.5	1.3	3203.3	1.5	3196.4	4.2	3201.8	3.7
3207.7	6.6	3206.4	0.6	3201.8	2.3	3205.9	1.4
3207.8	5.6	3208.5	5.1	3206.0	0.6	3206.0	2.8
3208.0	3.8	3209.3	8.5	3208.9	0.9	3207.9	3.4
3216.3	3.8	3215.2	14.7	3212.4	15.5	3213.1	6.7
3216.4	4.3	3217.5	6.0	3214.1	11.6	3214.6	6.4
3216.9	4.2	3218.3	4.6	3220.2	0.8	3217.1	12.4

Table S5 continued from previous page

$(PhOH)_3$, conf. 1		$(PhOH)_3$, conf. 2		$(PhOH)_3$, conf. 3		$(PhOH)_3$, conf. 4	
ω, cm^{-1}	I, km/mol	ω, cm^{-1}	I, km/mol	ω, cm^{-1}	I, km/mol	ω, cm^{-1}	I, km/mol
3223.1	10.3	3223.7	11.4	3220.5	24.7	3223.3	14.0
3223.3	8.0	3224.5	10.6	3222.7	12.5	3224.0	13.0
3223.4	8.5	3225.9	11.0	3228.6	8.9	3227.7	9.2
3230.9	6.8	3232.1	1.7	3232.4	5.5	3232.2	10.0
3231.1	8.7	3232.2	20.5	3232.5	14.5	3232.7	10.2
3231.3	8.5	3233.1	7.2	3238.0	2.0	3233.7	6.1
3625.5	16.3	3627.0	578.8	3657.3	330.3	3617.7	437.7
3670.4	826.5	3693.8	977.8	3694.7	907.9	3678.0	1001.8
3671.7	816.8	3823.6	148.0	3858.7	68.3	3722.9	539.7
